# *Chromobacterium* Biopesticide Exposure Does Not Select for Resistance in *Aedes* Mosquitoes

**DOI:** 10.1128/mbio.00480-23

**Published:** 2023-04-05

**Authors:** Cecilia Springer Engdahl, Eric P. Caragata, Mihra Tavadia, George Dimopoulos

**Affiliations:** a W. Harry Feinstone Department of Molecular Microbiology and Immunology, Bloomberg School of Public Health, Johns Hopkins University, Baltimore, Maryland, USA; b Florida Medical Entomology Laboratory, Department of Entomology & Nematology, Institute of Food & Agricultural Sciences, University of Florida, Vero Beach, Florida, USA; Washington University in St. Louis School of Medicine

**Keywords:** mosquito, *Aedes aegypti*, dengue, biopesticide, *Chromobacterium* Csp_P, selection, insecticide resistance, RNA-Seq, fitness

## Abstract

Developing effective tools to control mosquito populations is essential for reducing the incidence of diseases like malaria and dengue. Biopesticides of microbial origin are a rich, underexplored source of mosquitocidal compounds. We previously developed a biopesticide from the bacterium Chromobacterium sp. Panama that rapidly kills vector mosquito larvae, including Aedes aegypti and Anopheles gambiae. Here, we demonstrate that two independent Ae. aegypti colonies exposed to a sublethal dose of that biopesticide over consecutive generations persistently exhibited high mortality and developmental delays, indicating that resistance did not develop during the study period. Critically, the descendants of biopesticide-exposed mosquitoes experienced decreased longevity and did not display increased susceptibility to dengue virus or decreased susceptibility to common chemical insecticides. Through RNA sequencing, we observed no link between biopesticide exposure and the increased activity of xenobiotic metabolism and detoxification genes typically associated with insecticide resistance. These findings indicate that the Chromobacterium biopesticide is an exciting, emerging mosquito control tool.

## INTRODUCTION

There are more than 3,600 different species of mosquito on the planet, and although just a small percentage are implicated in diseases of medical importance, their impact on human health is high (1), resulting in tens of millions of infections and millions of deaths each year (2). Key mosquito vector species are involved in the transmission of the pathogens responsible for diseases such as malaria, dengue, West Nile fever, and filariasis. Risks associated with these diseases are typically mitigated by controlling populations of these mosquito species, most commonly by targeting adults via spraying with chemical insecticides and the elimination of larval mosquito habitats. Common classes of insecticides used in mosquito control, such as pyrethroids and organophosphates, are highly effective killers of mosquitoes. However, over-reliance on these chemicals in areas of disease endemicity has led to the development of resistance in many mosquito populations (3). Resistance can be mediated by various distinct mechanisms, including target site insensitivity, metabolic enzyme-mediated detoxification, and penetration resistance (4). Resistance poses major problems for mosquito abatement because it leads to reductions in intervention efficacy and, consequently, increased risk of disease (5).

The global trend toward an increasing prevalence of insecticide resistance and the continued high burden of disease caused by mosquito-borne pathogens have prompted scientists to develop many novel and innovative approaches for controlling mosquito populations or limiting the transmission of medically important pathogens transmitted by mosquitoes (6, 7). Several of these approaches have been utilized successfully in large-scale interventions, including use of the bacterial endosymbiont Wolbachia pipientis to eliminate mosquito populations (8, 9) or to replace a pathogen-susceptible mosquito population with a pathogen-resistant population (10, 11). Another example involves the use of dominant lethal genetics and mosquito transgenesis to suppress target populations (12). Other approaches being explored in the laboratory or semifield trials include the use of conditionally lethal baits that contain double-stranded RNAs to exploit the mosquito RNA interference immune pathway and knock down the expression of mosquito genes essential for development or reproduction (13). Another promising and versatile option involves CRISPR/Cas9-mediated gene drive, which offers a rapid and effective means of pushing lethal or antipathogen genes into mosquito populations (14, 15). These approaches differ greatly in scope from historical mosquito control formats but offer great potential for use in integrated pest management.

In addition, scientists are continuing to search for novel insecticidal compounds that utilize unique mechanisms to kill mosquitoes, given that it is highly likely that such compounds will still be effective at killing mosquito populations that are already resistant to current insecticides. These compounds include biopesticides, which are insecticides of biological origin. Biopesticides can be derived from many sources, including natural products isolated from plants or microorganisms that can be metabolites, proteins, or other small molecules. Successful examples of biopesticides used in mosquito control include Lysinibacillus sphaericus and Bacillus thuringiensis subsp. *israelensis* (16, 17), which have high specificity and are very effective at killing mosquito larvae via gut-lysing crystal proteins. Other examples include entomopathogenic fungi that possess natural mosquitocidal activity, such as Beauveria bassiana and Metarhizium anisopliae (18, 19). Bed nets can be impregnated with these fungi to provide an extra layer of protection from mosquito bites (20), and the fungi can also be genetically modified to produce insecticidal proteins that lead to more rapid mosquito death (21). These studies suggest that microorganisms represent a valuable resource when it comes to mosquito biopesticide development (22, 23). Critically, the mosquito gut naturally harbors dozens or even hundreds of microorganisms (24, 25), each capable of producing numerous compounds and small molecules that may have mosquitocidal activity.

Another emerging mosquito control tool is a biopesticide based on the bacterium Chromobacterium species Panama (Csp_P) (class, *Betaproteobacteria*; family, *Neisseriaceae*). This bacterium was first isolated from Aedes aegypti mosquitoes collected in Panama (26). Interestingly, sublethal doses of Csp_P induce two distinct anti-pathogen effects in mosquitoes (27). The first is mediated by the depsipeptide romidepsin, which restricts infection of Plasmodium falciparum, the human malarial parasite, in Anopheles gambiae mosquitoes (28). The second is caused by an aminopeptidase that degrades the envelope protein of dengue virus (DENV), reducing the virus’s ability to infect Ae. aegypti (29). Live Csp_P cultures have strong mosquitocidal properties, with Csp_P exposure leading to rapid death of adults and larvae from key mosquito vector species, including A. gambiae and Ae. aegypti (27, 30, 31). We previously developed a biopesticide bait based on a crude extract of nonlive Csp_P biofilm (32). These air-dried Csp_P baits are inexpensive and easy to prepare, and a critical feature is their low effective dosage, with an estimated 50% lethal dose (LD_50_) of 11.35 mg/liter of larval aquatic habitat. The Csp_P biopesticide has an extensive shelf life and has proved to be a highly effective insecticide against several important mosquito vector species under both laboratory and semifield conditions. These are all promising traits for a biopesticide.

A key aspect of mosquito control interventions involving chemical insecticides or biopesticides is to assess potential off-target effects that result from suboptimal or long-term usage of the product. Long-term and multigenerational exposure of mosquitoes to insecticides can promote resistance (33–35), it can also lead to changes in life history traits (36, 37), and it can even alter susceptibility to pathogen infection (38). To explore the potential impact of a mosquito control strategy employing the Csp_P biopesticide, we have examined the effects of exposure to the Csp_P biopesticide over 9 to 10 generations in two populations of the mosquito Ae. aegypti, an important vector of the dengue, chikungunya, yellow fever, and Zika viruses. We were interested in determining whether resistance would arise if Ae. aegypti larvae were repeatedly exposed to a dosage of the biopesticide that is too low to kill the entire population, and whether the molecular mechanisms of resistance would be similar to those seen for conventional insecticides (39, 40). In addition, we wanted to examine whether this multigenerational exposure to the Csp_P biopesticide had adverse consequences for mosquito fitness or susceptibility to conventional insecticides and whether it altered susceptibility to infection with DENV.

To address these research questions, we exposed larvae from two different laboratory colonies of Ae. aegypti, originally isolated from Puerto Rico and Singapore, to the Csp_P biopesticide for nine consecutive generations and observed consistent levels of mortality and delays in development across all generations of the selection regimen, compared to nonexposed sibling lines. Postselection fitness assays revealed that Csp_P biopesticide exposure did not have consistent impacts on fecundity, fertility, or wing length, but the longevity of males, sugar-fed females, and blood-fed females was reduced by biopesticide exposure for both Ae. aegypti colonies. Using RNA sequencing (RNA-Seq), we generated the transcriptomes of exposed and nonexposed mosquitoes. While biopesticide exposure did induce transcriptomic changes, we saw few similarities between the two mosquito colonies, but we also did not observe any evidence that exposure increased the expression of gene classes typically linked to insecticide resistance. Similarly, we found no evidence that exposure to the Csp_P biopesticide led to increased susceptibility to dichlorodiphenyltrichloroethane (DDT), deltamethrin, bendiocarb, or malathion after mosquitoes were exposed to these insecticides following the World Health Organization (WHO) adult insecticide susceptibility protocol. Finally, via experimental oral infection, we demonstrate that biopesticide-exposed mosquitoes were not more susceptible to infection with DENV-2.

## RESULTS

### Biopesticide efficacy after multigenerational exposure of Ae. aegypti larvae.

**FIG 1:**
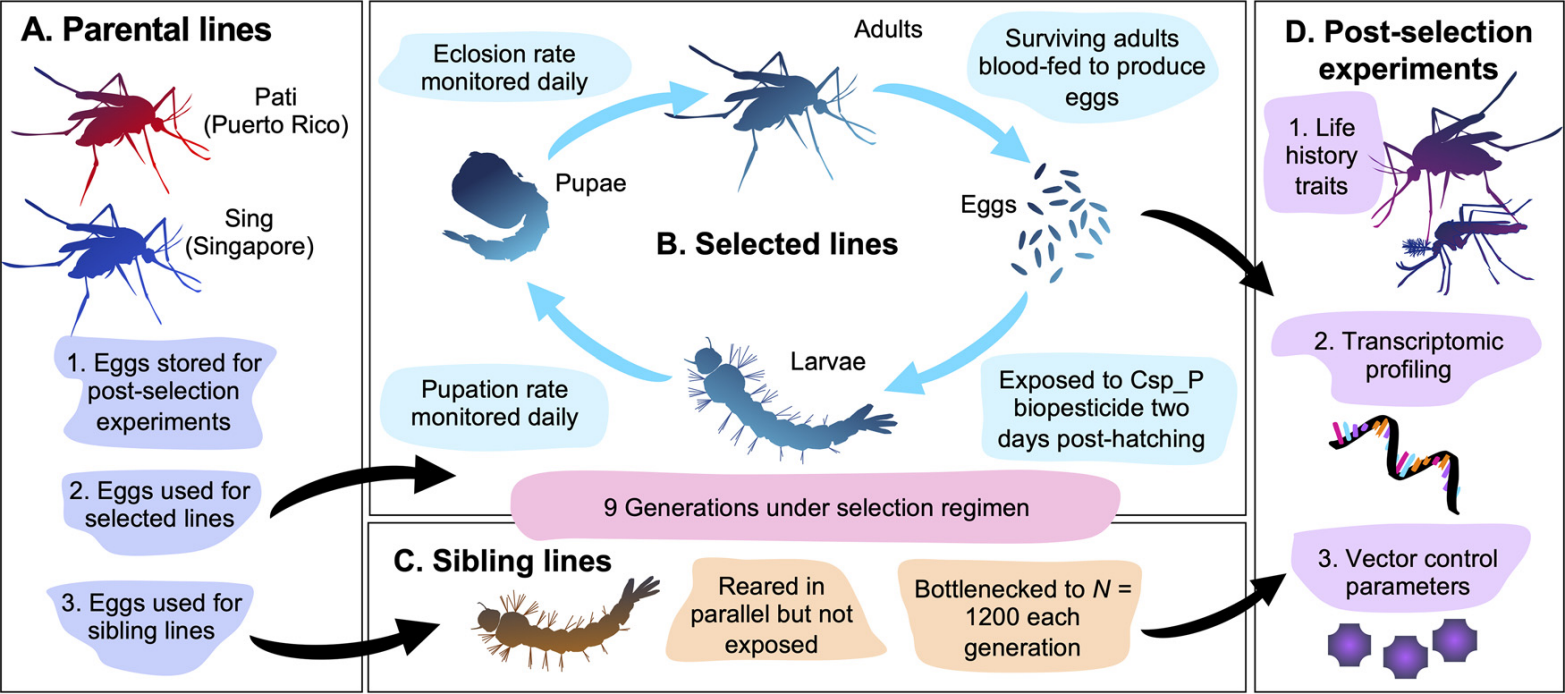
Schematic overview of selection process and postselection experiments. (A) Parental eggs from the Pati (Patillas, Puerto Rico) and Sing (Singapore) Ae. aegypti populations were used to generate (B) selected (Csp_P biopesticide-exposed) and (C) sibling (bottlenecked but not exposed) lines. The selection regimen was enacted for nine generations, and during this time, developmental parameters were monitored daily. (D) After selection, life history traits were assayed (fecundity, fertility, wing length, and longevity), the transcriptome was profiled using RNA sequencing (RNA-Seq). The mosquitoes were exposed to common chemical insecticides, as well as DENV-2, in order to evaluate the consequences of multigenerational exposure to the Csp_P biopesticide.

We exposed larvae from two different Ae. aegypti colonies, Pati (origin Puerto Rico) and Sing (origin Singapore), to the Csp_P biopesticide via food baits for nine consecutive generations (Fig. 1). In each generation, we measured the mean pupation time, mean eclosion time, and eclosion rate for biopesticide-selected mosquitoes and also for mosquitoes from their sibling counterpart lines, which were not exposed to the biopesticide but were reared in parallel and bottlenecked to 1,200 larvae/generation (Data Set S1).

10.1128/mbio.00480-23.1 DATA SET S1 Developmental data associated with the selection regime. Download Data Set S1, XLSX file, 0.3 MB. Copyright © 2023 Engdahl et al. 2023 Engdahl et al. https://creativecommons.org/licenses/by/4.0/ This content is distributed under the terms of the Creative Commons Attribution 4.0 International license.

**FIG 2:**
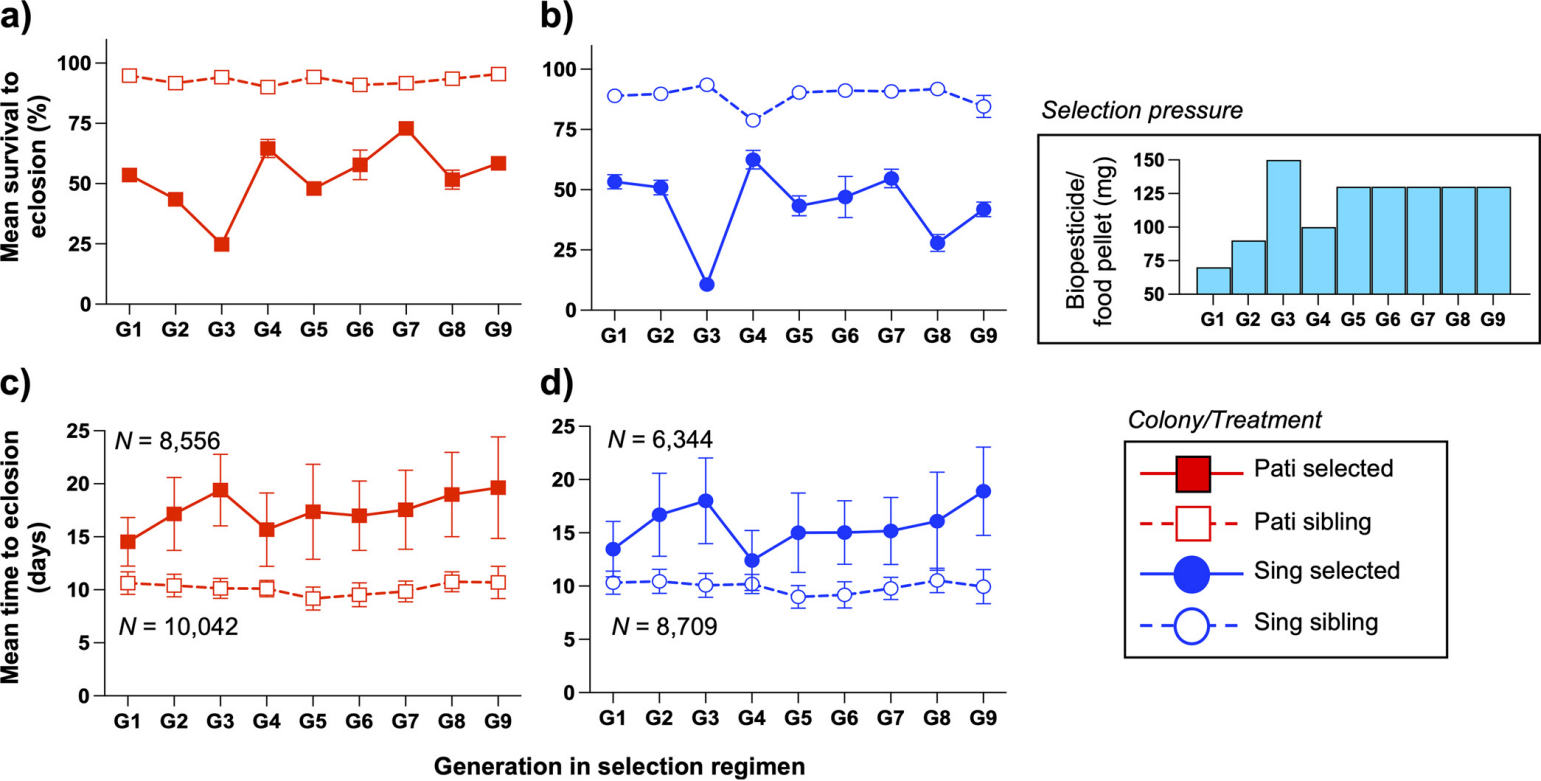
Multigenerational exposure to the Csp_P biopesticide consistently induces high mortality and developmental delays. (a, b) For each generation within the selection regimen, developmental parameters were monitored and used to calculate the survival rate for both the Pati (a) and Sing (b) lines. Our data showed significant differences in survival between the selected and sibling lines in every generation. Although the scale of those differences varied between generations, these data were not indicative of resistance developing over the course of the experiment. (c, d) Mean time to eclosion for Pati (c) and Sing (d) mosquitoes was significantly longer for selected lines than for sibling lines, with the delay equating to approximately 5 to 7 days. The consistent occurrence of mortality effects and developmental delays suggested that neither colony was becoming resistant to the biopesticide after nine generations of selection. The selection pressure box details the dose of biopesticide per bait in each generation. Dosage and efficacy were varied due to batch-to-batch variation effects. Each data point represents the overall mean per treatment per generation. The bars represent the standard error of the mean.

During each day of the selection regimen, we recorded the number of newly eclosed adult mosquitoes produced by each larval tray. These data were used to calculate overall eclosion rates, which served as a proxy for survival rates after exposure to the Csp_P biopesticide. Across the nine generations of selection, we recorded overall eclosion rates of 92.98% for the Pati sibling line, 52.81% for the Pati selected line, 89.47% for the Sing sibling line, and 42.90% for the Sing selected line. The survival rate was compared between selected and sibling lines across generations for both the Pati (Fig. 2a) and Sing colonies (Fig. 2b). For both colonies, we observed consistently lower mortality in the selected lines than in the sibling lines (two-way analysis of variance [ANOVA]: Pati – *F *= 1,216,710, *P < *0.0001; Sing – *F *= 645,079, *P < *0.0001), and we also observed that the survival rate of the selected lines fluctuated each generation in accordance with the dose of the biopesticide that was used (two-way ANOVA: Pati – *F* = 12,277, *P* < 0.0001; Sing – *F* = 4,598, *P* < 0.0001).

Mean pupation time was consistently delayed by exposure to the biopesticide across the nine generations of selection. Across all generations, the mean time to pupation was 8.12 days for Pati sibling mosquitoes and 15.42 days for Pati selected mosquitoes. For the Sing colony, the mean pupation time was 7.97 days for the sibling line and 13.94 days for the selected line. Mean pupation varied significantly as a result of exposure to the Csp_P biopesticide for the Pati and Sing colonies (two-way ANOVA: Pati – *F* = 37,092, *P* < 0.0001; Sing – *F *= 21,386, *P* < 0.0001) and also varied across generations, depending on the biopesticide dosage (two-way ANOVA: Pati – *F* = 245.5, *P* < 0.0001; Sing – *F* = 259.6, *P* < 0.0001).

Similarly, the mean time to eclosion was delayed by exposure to the biopesticide for both the Pati (Fig. 2c) and Sing colonies (Fig. 2d), with the extent of the delay being correlated with the biopesticide dosage employed. Overall, the mean time to eclose was 10.14 days for Pati sibling mosquitoes and 17.48 days for Pati selected mosquitoes. For the Sing colony, the mean eclosion time was 9.94 days for the sibling line and 15.65 days for the selected line. For both colonies, the mean eclosion varied significantly because of exposure to the Csp_P biopesticide (two-way ANOVA: Pati – *F *= 33,684, *P < *0.0001; Sing – *F *= 17,070, *P < *0.0001). We also observed significant differences across generations (two-way ANOVA: Pati – *F *= 244.6, *P < *0.0001; Sing – *F *= 261.4, *P < *0.0001), but the data did not support the notion that faster time to eclosion occurred as the number of generations of exposure to the biopesticide increased.

**FIG 3:**
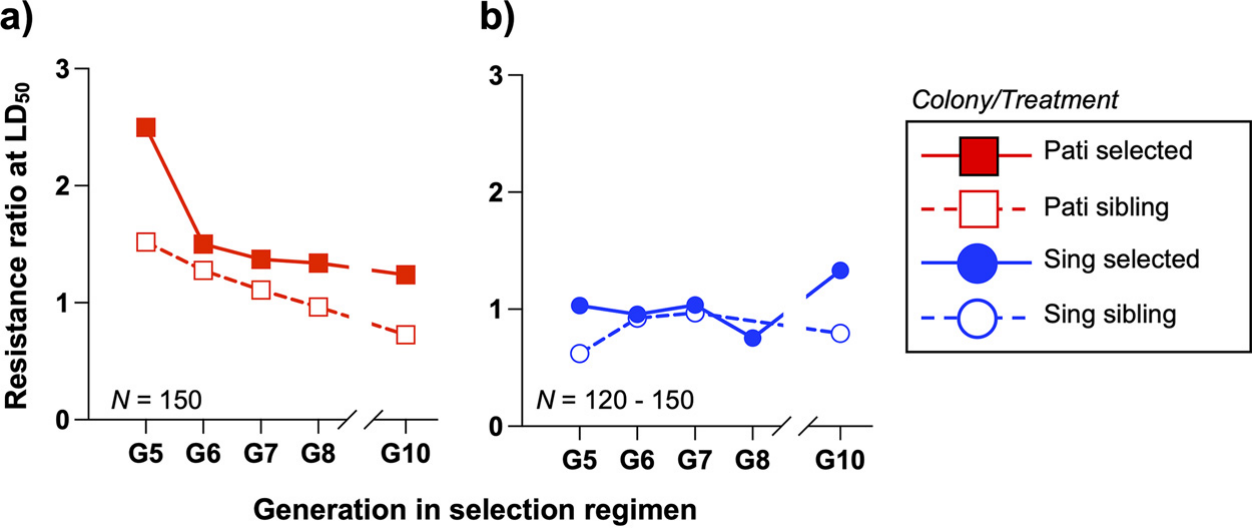
Multigenerational exposure to the Csp_P biopesticide does not induce resistance in selected lines. Resistance ratios for Pati (a) and Sing (b) mosquito lines were calculated by comparing the 50% lethal dose (LD_50_) of Csp_P biopesticide for selected versus parental mosquito lines (solid lines) and sibling versus parental lines (dashed lines). Comparison of these data via simple linear regression revealed that the resistance ratios for the selected and sibling lines for both colonies changed in the same way across generations and provided no indication that resistance had arisen in the two selected lines, with a higher resistance ratio value indicating greater resistance than the parental line.

Resistance ratios were calculated for sibling and selected lines from the Pati (Fig. 3a) and Sing colonies (Fig. 3b) by comparing LD_50_ values for those lines against their respective parental lines. These values were calculated between G_5_ and G_10_ during the selection regimen. Measurements were not made on G_9_ mosquitoes because there were insufficient numbers available after allocation for the fitness assays. The resistance ratio values were plotted, and the slopes of the sibling and selected lines were compared using simple linear regression (SLR). We observed that for both the Pati (SLR: *F *= 0.5295, *P = *0.4942) and Sing colonies (SLR: *F *= 0.1906, *P = *0.6850), there was no significant effect of Csp_P biopesticide exposure on the resistance ratio, indicating that there was no evidence that either of the selected lines became more tolerant to the biopesticide over time.

### Impact of multigenerational biopesticide exposure on Ae. aegypti life history traits.

We assessed multiple life history parameters for the selected and sibling lines after nine or ten generations of selection to determine whether multigenerational exposure to the Csp_P biopesticide affected mosquito fitness (Data Set S2). Cohorts of G_3_ parental line mosquitoes were included in all assays to assess the impact of selection and bottlenecking experienced by the other two lines.

10.1128/mbio.00480-23.2 DATA SET S2 Data from post-selection fitness assays. Download Data Set S2, XLSX file, 0.04 MB. Copyright © 2023 Engdahl et al. 2023 Engdahl et al. https://creativecommons.org/licenses/by/4.0/ This content is distributed under the terms of the Creative Commons Attribution 4.0 International license.

**FIG 4:**
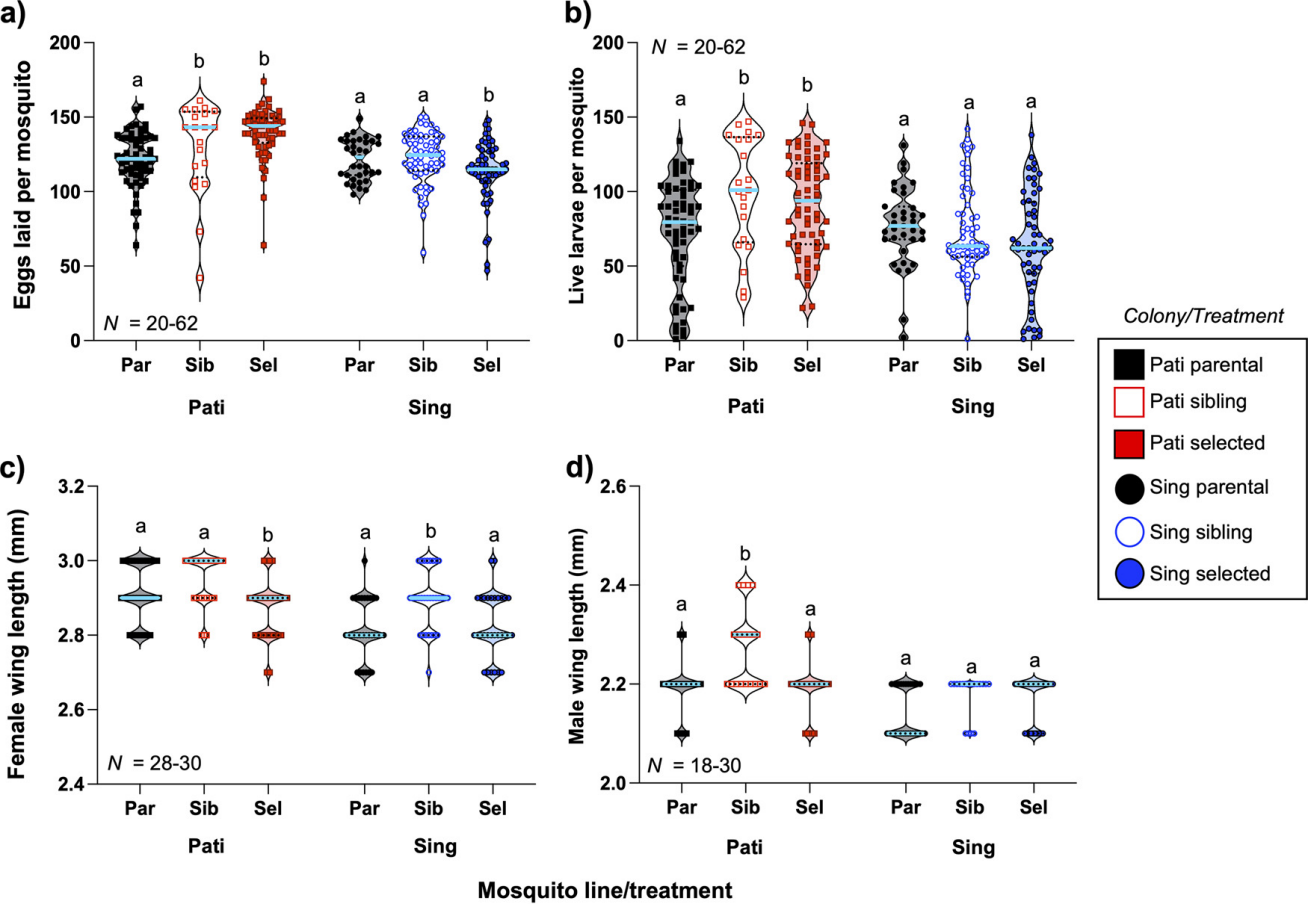
Multigenerational exposure to the Csp_P biopesticide does not consistently reduce fecundity, fertility, or body size. Life history traits were assessed for parental, sibling, and selected lines from both colonies postselection and are presented as violin plots. (a) Fecundity was assessed after a mouse blood meal. For Pati mosquitoes, fecundity was significantly higher for the sibling and selected lines than for the parental line. For Sing mosquitoes, fecundity was significantly reduced in the selected line compared to both other lines. (b) Fertility was assessed for mosquitoes that had laid eggs in the fecundity assay. As for fecundity, Pati fertility was significantly higher in the sibling and selected lines. We observed no significant differences between the three Sing lines. (c, d) Wing length was measured for an independent cohort of female (c) and male (d) mosquitoes. For Pati females, the selected line had significantly shorter wings. For Sing females, the sibling line had larger wings than the other two lines. Pati sibling males had larger wings than did the other two lines. We observed no differences in wing length among Sing males. These data indicate that multigenerational exposure to the Csp_P biopesticide does induce fitness costs linked to these parameters, but we saw no consistent pattern of fitness effects between the Pati and Sing colonies. Each data point represents a measurement from a single mosquito. Solid blue lines indicate treatment medians. Dashed blue lines indicate upper and lower quartiles. The letters above the data sets indicate statistical groups as determined by Kruskal-Wallis analysis of variance (ANOVA). Par, parental line; Sel, selected line; Sib, sibling line.

We observed that exposure to the biopesticide had distinct effects on the fecundity of Pati and Sing mosquitoes (Fig. 4a). For the Pati colony, the median number of eggs laid per mosquito was 122 for the parental line, 143 for the sibling line, and 144 for the selected line. For the Sing colony, the median number of eggs laid per female were 123 for the parental line, 125 for the sibling line, and 115 for the selected line. Multiple linear regression (MLR) revealed that the selection regimen had a significant effect on fecundity (MLR; Treatment[selected], *t *= 5.25, *P < *0.0001), but that effect differed between the Pati and Sing colonies (MLR; Colony × Treatment[selected], *t *= 5.01, *P < *0.0001), with increased fecundity observed in the selection treatment for Pati mosquitoes and decreased fecundity for Sing mosquitoes. These effects were confirmed via Dunn’s test. No significant effects were observed for colony, sibling treatment, or colony × sibling interaction.

In terms of fertility (Fig. 4b), the Pati parental, sibling, and selected lines produced median numbers of live larvae of 80, 101, and 94, respectively; for the Sing colony, the median numbers of live larvae were 77, 64, and 62, respectively. Multiple linear regression revealed no significant impact of colony on fertility (MLR; Colony, *t *= 0.57, *P = *0.5718), but there were significant effects for sibling (MLR; treatment[sibling], *t *= 3.23, *P = *0.0014) and selected treatments (MLR; treatment[selected], *t *= 3.14, *P = *0.0019), and for both colony × treatment interaction terms (MLR; Colony × Treatment(sibling), *t *= 2.789, *P = *0.0057; Colony × Treatment(selected), *t *= 3.33, *P = *0.001). Dunn’s tests indicated that the fertility was significantly higher for the Pati sibling and selected lines than for the parental line but indicated no significant differences related to treatment for the three Sing lines.

We observed that the average wing length for female mosquitoes was slightly longer for Pati mosquitoes (2.91 mm) than for Sing mosquitoes (2.85 mm) (Fig. 4c). Multiple linear regression indicated significant differences in female wing length between colonies (MLR; Colony, *t *= 4.72, *P < *0.0001) and as a result of selection treatment (MLR; Treatment[sibling], *t *= 2.28, *P = *0.0238). Dunn’s test indicated that selection effects were associated with a slight decrease in wing length for the Pati selected line (average length = 2.87 mm) compared to the parental line (2.91 mm). No significant effects were observed for the Sing colony. Male wing length (Fig. 4d) also differed significantly between colonies (MLR; Colony, *t *= 3.27, *P = *0.0013), with slightly longer average wing lengths observed among the Pati males (2.21 mm versus 2.16 mm). Significant effects were also observed for the sibling treatment (MLR; Treatment[sibling], *t *= 5.12, *P < *0.0001), and the colony × sibling treatment interaction (MLR; Colony × Treatment[sibling], *t *= 2.13, *P = *0.0349). These effects were driven by larger males in the Pati sibling treatment (average wing length = 2.27 mm). Thus, there were no significant effects on male wing length associated with biopesticide exposure.

**FIG 5:**
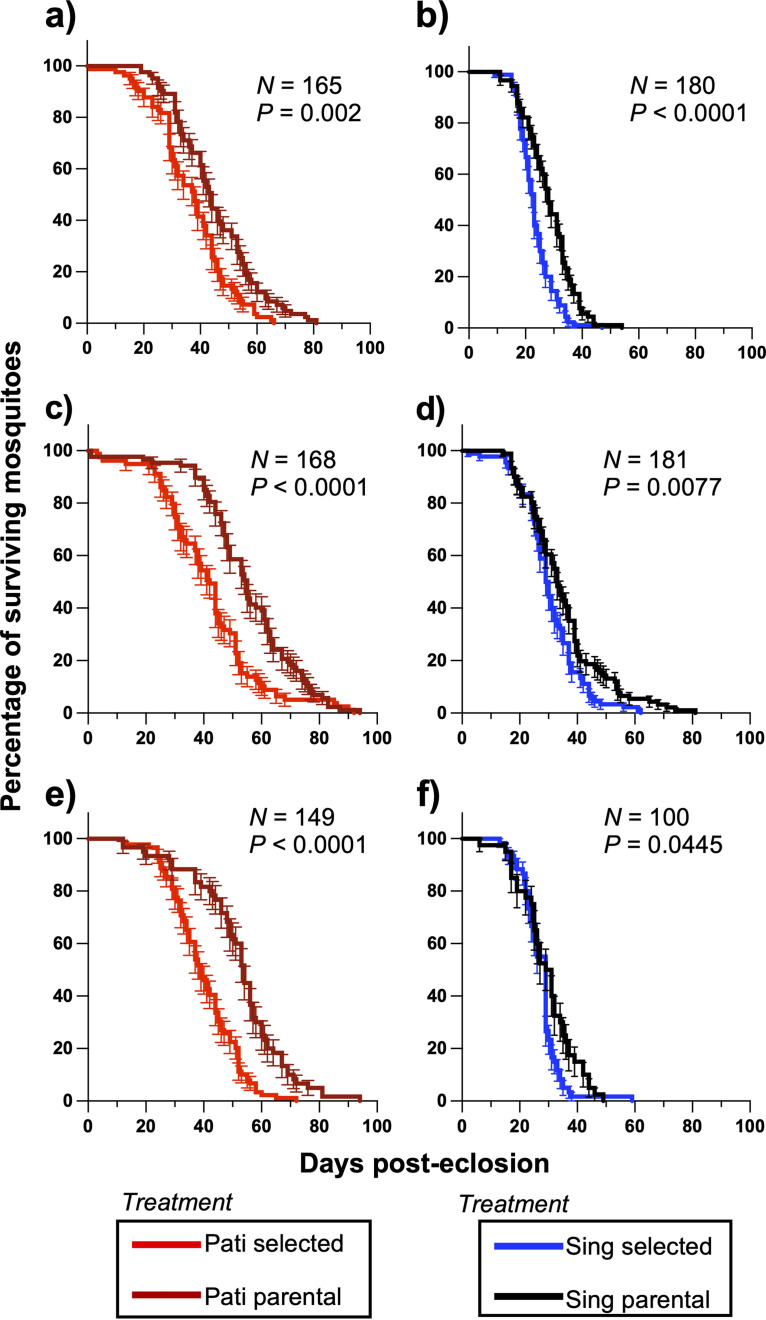
Multigenerational exposure to the Csp_P biopesticide reduces adult longevity. Longevity of selected and parental lines was compared postselection by counting and removal of dead sugar-fed females (a, b), sugar-fed males (c, d), and blood-fed females (e, f) every 1 to 2 days. Comparison of parental and selected line data with Mantel-Cox tests revealed that prior biopesticide exposure significantly decreased adult mosquito longevity in all assays. The bars represent the standard error of the mean.

Longevity assays revealed that multigenerational exposure to the Csp_P biopesticide led to a significantly decreased survival time for selected mosquitoes compared to the parental mosquitoes. This effect was observed for sugar-fed female mosquitoes (Fig. 5a and b; Mantel-Cox; Pati – χ^2^ = 13.59, hazard ratio [HR] = 1.88, *P = *0.0002; Sing – χ^2^ = 22.71, HR = 2.26, *P < *0.0001), for sugar-fed male mosquitoes (Fig. 5c and d; Mantel-Cox; Pati – χ^2^ = 18.52, HR = 2.11, *P < *0.0001; Sing – χ^2^ = 7.11, HR = 1.54, *P = *0.0077), and for blood-fed female mosquitoes (Fig. 5e and f; Mantel-Cox; Pati – χ^2^ = 34.93, HR = 2.98, *P < *0.0001; Sing – χ^2^ = 4.04, HR = 1.57, *P = *0.0445). HRs associated with selection varied from 1.57 for blood-fed Sing females to 2.98 for blood-fed Pati females, indicating a 1.57- to 2.98-fold increase in risk of mortality in adult mosquitoes that were exposed to the biopesticide as larvae.

### Transcriptomic changes imposed by multigenerational Csp_P biopesticide exposure.

**FIG 6:**
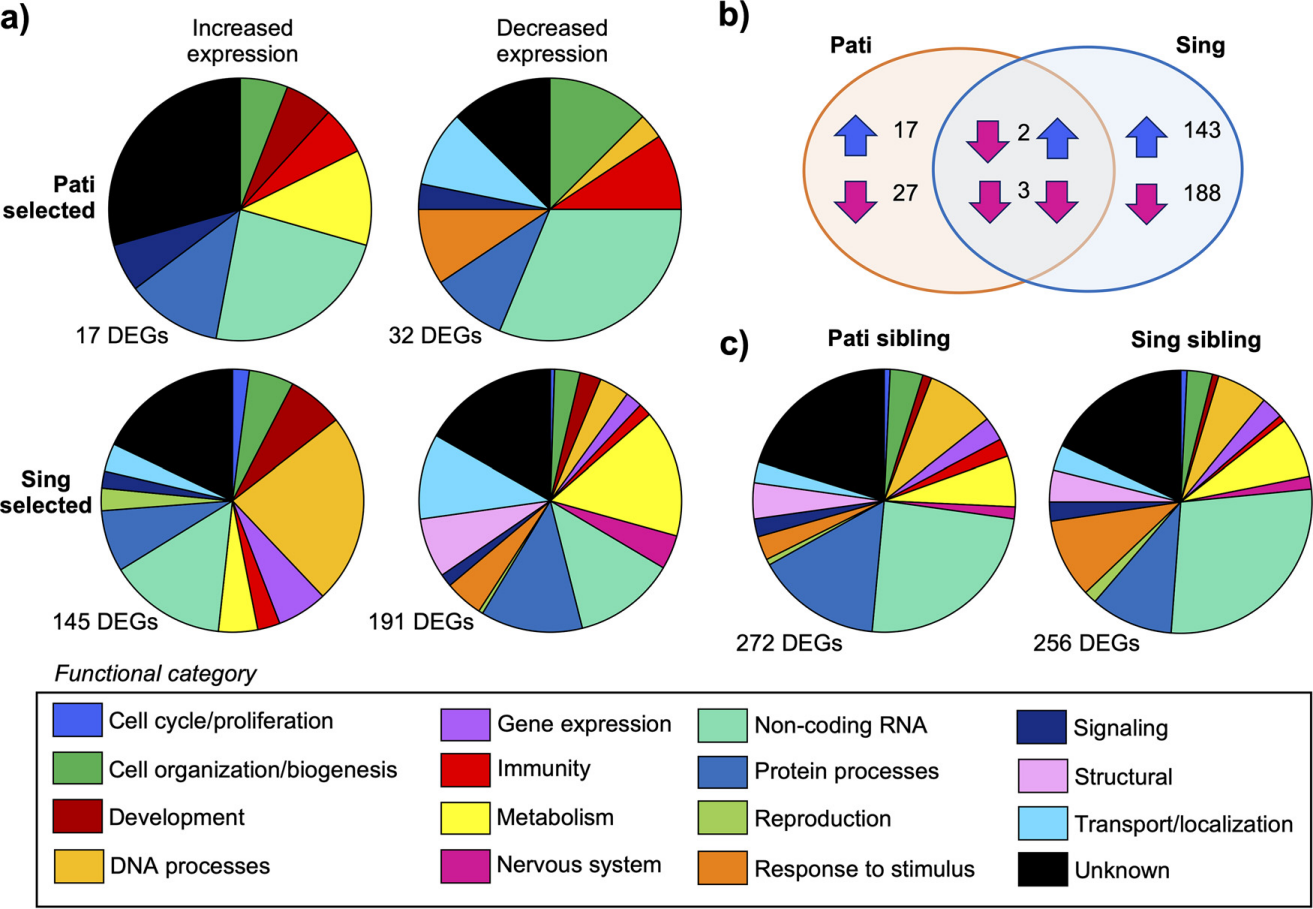
Multigenerational Csp_P biopesticide exposure is not associated with a clear molecular signature of resistance. The postselection transcriptomic profiles of parental, selected, and sibling line mosquitoes from the Pati and Sing colonies were generated using RNA-Seq. Differentially expressed genes (DEGs) were identified using DESeq2 in R, and these genes were annotated using the VectorBase, InterPro, UniProt, and FlyBase databases. Pie charts depict the percentage of DEGs allocated to 16 different functional categories. (a) We observed differences in the scale and scope of DEGs associated with Pati selected (versus Pati sibling/parental) and Sing selected (versus Sing sibling/parental) mosquitoes, indicating that the two colonies had very different molecular responses to Csp_P biopesticide exposure. (b) A Venn diagram of the 380 DEGs identified between both selected lines and their sibling counterparts indicated that only 5 DEGs were shared between the colonies, and only 3 of those DEGs were affected in the same manner. Blue arrows indicate increased expression, and pink arrows indicate decreased expression. (c) Comparison of the transcriptomes of the two sibling lines revealed 528 DEGs, with similar distributions across functional categories. This observation suggests that the two mosquito colonies in our experiments had similar patterns of gene expression overall, but it appears likely that they employ different networks of genes to achieve those ends.

We used RNA-Seq to examine the impact of multigenerational exposure to the Csp_P biopesticide on Ae. aegypti gene expression. Our sequencing compared the parental, sibling, and selected lines from both the Pati and Sing colonies and produced 804,594,144 reads (Data Set S3), of which 747,197,743 reads (92.87%) were successfully mapped to the Ae. aegypti genome. For both colonies, we focused our analysis on the differences between the selected lines compared to the sibling and parental lines (Fig. 6a). Our intent was to identify whether genes that could be involved in insecticide resistance, the stress response, xenobiotic metabolism, or detoxification experienced changes in expression after Csp_P biopesticide exposure. Additionally, we were interested in identifying genes that might explain the delays in development seen in exposed mosquitoes and in seeing whether the nonlive biopesticide induced an immune response. For Pati mosquitoes, we observed 17 genes with a higher expression as a result of selection and a further 32 genes with a decreased expression. The scale and scope of the transcriptional response was greater in Sing mosquitoes; a total of 145 genes showed an increased expression in the selected line, and 191 genes showed a decreased expression. Comparing these lists of genes (Data Set S4) led to the observation that there were few common responses to Csp_P biopesticide exposure, since only five DEGs were affected for both colonies (Fig. 6b). Of those, four were long noncoding RNAs (AAEL021984, AAEL022280, AAEL025002, and AAEL020201). The fifth gene (AAEL026694) was a protein inhibitor of activated STAT 2 (*pias2*), a gene predicted to regulate the JAK/STAT immune pathway; however, the effects of selection were inconsistent, showing an increase in expression in the Sing colony and a decrease in the Pati colony.

10.1128/mbio.00480-23.3 DATA SET S3 Raw RNA-Seq readcounts. Download Data Set S3, XLSX file, 1.7 MB. Copyright © 2023 Engdahl et al. 2023 Engdahl et al. https://creativecommons.org/licenses/by/4.0/ This content is distributed under the terms of the Creative Commons Attribution 4.0 International license.

10.1128/mbio.00480-23.4 DATA SET S4 DEGs (Selected vs Sibling/Parental). Download Data Set S4, XLSX file, 0.05 MB. Copyright © 2023 Engdahl et al. 2023 Engdahl et al. https://creativecommons.org/licenses/by/4.0/ This content is distributed under the terms of the Creative Commons Attribution 4.0 International license.

In the absence of broad commonalities between the colonies, we chose instead to independently identify interesting transcriptional changes between the sibling, parental, and selected lines for each colony (Table 1). In the Pati comparison, there were only 49 DEGs. Fourteen of these genes were noncoding RNAs, with this being the most common functional category. Notable genes with higher expression after biopesticide exposure included a histone methyltransferase complex subunit ASH2 (AAEL022498), which likely plays a regulatory role in embryo development; a carboxylic ester hydrolase (AAEL002391), which could be involved in xenobiotic metabolism; and leucine-rich immune protein 25 (AAEL009792).

**TABLE 1:** Notable differentially expressed genes associated with Csp_P biopesticide exposure^*a*^

Gene ID	Gene name	log2FC	Adjusted *P*	Functional category	Likely function
Pati: upregulated DEGs					
AAEL022406	Actin-binding cytoskeletal protein	1.3428	0.0469	Cell organization/biogenesis	Structural cytoskeletal protein
AAEL022498	Histone methyltransferase complex subunit ASH2	2.4522	0.0173	Development	Regulation of transcription during pattern forming/development
AAEL009792	Leucine-rich immune protein 25 (LRIM25)	1.3689	0.0429	Immunity	Innate immune protein
AAEL002391	Carboxylic ester hydrolase	1.5219	0.0030	Metabolism	Xenobiotic metabolism
Pati: downregulated DEGs					
AAEL009036	Immunoglobulin-like domain-containing protein	−9.4675	0.0037	Cell organization/biogenesis	Recognition/binding
AAEL002307	Leucine-rich transmembrane protein	−2.0711	0.0024	Cell organization/biogenesis	Varied functions possible
AAEL026694	PIAS2	−1.9811	0.0028	Immunity	JAK/STAT regulator/hedgehog regulator
AAEL021557	Poor Imd response upon knock-in (pirk)	−1.3666	0.0245	Immunity	IMD regulator
AAEL015294	Serine type endopeptidase	−2.1268	0.0244	Protein processes	Proteolysis
AAEL012809	Peptidylprolyl isomerase	−2.6681	0.0245	Protein processes	Facilitates protein-chaperone interaction
AAEL006424	D7 protein	−8.9775	7.47E−10	Response to stimulus	Odorant binding protein
AAEL000340	Cytochrome p450	−3.4627	0.0002	Response to stimulus	Detoxification
AAEL013352	Lethal (2) essential for life, l2efl	−1.2711	0.0212	Response to stimulus	Heat shock protein
AAEL022056	Vacuolar protein pump	−2.6310	0.0001	Transport/localization	Facilitates proton transport
Sing: upregulated DEGs					
AAEL027716	Actin-related protein 2/3 complex subunit 3	3.0182	0.0257	Cell organization/biogenesis	Regulation of actin filament polymerization
AAEL005956	Caspase (short) (CASPS16)	1.3750	6.41E−11	Development	Neuronal apoptosis during embryogenesis
AAEL022628	Female sterile (q) M3-like protein	1.2995	2.92E−7	Development	Eggshell protein
AAEL004130	Homeobox transcription factor, IND-like	3.0278	2.81E−5	Development	CNS development
AAEL027412	DDE family endonuclease	2.6516	0.0004	DNA processes	Digestion of DNA
AAEL026694	PIAS2	1.4085	0.0130	Immunity	JAK/STAT regulator/hedgehog regulator
AAEL011001	Periplasmic binding domain-containing protein	2.8970	0.0269	Immunity	Facilitates binding of bacterial periplasm
AAEL025170	*S*-Adenosylmethionine decarboxylase proenzyme (SamDC)	1.4587	1.90E−10	Immunity	Anti-bacterial defense
AAEL019903	CBM39 domain-containing protein	3.4934	0.0281	Immunity	Carbohydrate binding/microbe recognition
AAEL011305	Leucine-rich repeat protein	2.9419	0.0002	Protein processes	Protein-protein interactions
AAEL009642	Cathepsin B	1.5988	5.54E−10	Protein processes	Proteolysis
AAEL006138	Vitellogenin A1	1.9547	5.46E−10	Reproduction	Vitellogenesis
AAEL006563	Vitellogenic carboxypeptidase Precursor (VCP)	1.4857	0.0008	Reproduction	Vitellogenesis
AAEL021453	Female sterile (1) M3	1.4679	4.57E−8	Reproduction	Vitelline membrane integrity
AAEL018103	Vitellogenin domain-containing protein	1.3860	1.23E−14	Reproduction	Vitellogenesis
AAEL018316	CNM amide receptor	2.7036	0.0010	Signaling	G-protein coupled receptor signaling
AAEL009562	Zinc finger protein (SWIM type)	1.4125	0.0092	Unknown	Zinc finger protein with unknown function
Sing: downregulated DEGs					
AAEL004646	Actin	−1.6621	0.0002	Cell organization/biogenesis	Cytoskeleton formation
AAEL005293	Galectin (GALE8A)	−1.2578	6.72E−6	Cell organization/biogenesis	Cell-cell interactions
AAEL026278	Hemolymph juvenile hormone binding protein	−1.6144	5.39E−11	Development	Larval development
AAEL004987	Hemolymph juvenile hormone binding protein	−1.3325	8.09E−14	Development	Larval development
AAEL001323	Hemolymph juvenile hormone binding protein	−1.3027	2.34E−10	Development	Larval development
AAEL021586	Toll-like receptor protein	−4.7596	0.0161	Immunity	Immune signaling
AAEL009178	Gram-negative binding protein B4 (GNBPB4)	−1.6130	0.0088	Immunity	Bacteria recognition
AAEL000652	Gram-negative binding protein A2 (GNBPA2)	−1.3836	0.0314	Immunity	Bacteria recognition
AAEL013385	Brain chitinase and chia	−2.6676	0.0040	Metabolism	Chitin metabolism
AAEL011897	Chitin-binding protein	−2.1063	0.0001	Metabolism	Chitin metabolism
AAEL012426	Cytochrome *c* oxidase assembly protein cox15	−1.7757	0.0461	Metabolism	Heme biosynthesis
AAEL011406	Cytochrome *c* oxidase polypeptide	−1.7107	0.0015	Metabolism	Respiratory chain complex
AAEL004953	Elongation of very long-chain fatty acids protein	−2.3732	0.0290	Metabolism	Fatty acid synthesis
AAEL015313	Odorant binding protein OBP59	−1.7155	0.0026	Nervous system	Odorant binding
AAEL000124	Odorant-binding protein	−1.4857	1.12E−7	Nervous system	Odorant binding
AAEL001675	Clip-domain serine protease (CLIPA10)	−2.2375	0.0149	Protein processes	Proteolysis
AAEL019920	Trypsin	−1.9049	0.0027	Protein processes	Proteolysis
AAEL011553	Trypsin	−1.6530	0.0055	Protein processes	Proteolysis
AAEL021257	Serine protease	−1.5785	0.0019	Protein processes	Proteolysis
AAEL022646	Larval chymotrypsin-like protein	−1.3452	1.21E−15	Protein processes	Proteolysis
AAEL009166	Insect allergen-like	−4.5033	0.0435	Response to stimulus	Detoxification/xenobiotic metabolism
AAEL019761	Superoxide dismutase (Cu-Zn)	−2.2792	0.0008	Response to stimulus	Reduction of superoxides
AAEL007962	Glutathione *S*-transferase E4 (GSTE4)	−1.4579	3.92E−14	Response to stimulus	Detoxification/xenobiotic metabolism
AAEL014594	cytochrome P450 (CYP301A1)	−1.3242	0.0053	Response to stimulus	Detoxification
AAEL002309	Thioredoxin Peroxidase (TPX4)	−1.3136	2.03E−17	Response to stimulus	Antioxidant defense
AAEL005113	Carboxy/choline esterase α-esterase (CCEAE1A)	−1.2982	0.0003	Response to stimulus	Detoxification
AAEL008532	Carboxylic ester hydrolase	−2.3413	0.0011	Response to stimulus	Xenobiotic metabolism
AAEL000630	Crustacean cardioacceleratory peptide (ccap)	−3.2482	8.94E−6	Signaling	Signaling neuropeptide
AAEL022261	Cuticle protein	−4.5587	4.13E−65	Structural	Structural component of cuticle
AAEL024751	Collagen alpha	−3.4432	0.0177	Structural	Extracellular structural protein
AAEL002231	Cuticle protein	−2.4529	1.87E−16	Structural	Structural component of cuticle
AAEL023525	Chitin-binding peritrophin	−2.2539	0.0005	Structural	Component of peritrophic matrix
AAEL015119	Cuticle protein	−2.1643	0.0061	Structural	Structural component of cuticle
AAEL024660	Pupal cuticle protein	−1.6749	5.14E−6	Structural	Structural component of cuticle
AAEL012646	Chitin-binding protein	−1.6450	1.30E−6	Structural	Component of peritrophic matrix
AAEL013517	Pupal cuticle protein 78E	−1.5751	0.0248	Structural	Structural component of cuticle
AAEL006749	Insect cuticle protein	−1.5001	9.05E−6	Structural	Structural component of cuticle
AAEL000419	Cuticle protein	−1.6235	0.0001	Structural	Structural component of cuticle
AAEL017081	Cytochrome *c* oxidase copper chaperone	−1.7508	0.0030	Transport and localization	Copper ion transport in mitochondria
AAEL020775	Apolipoprotein D	−4.6857	0.0125	Transport and localization	Intracellular lipid transport
AAEL026174	Niemann-Pick type C-2	−4.5772	0.0249	Transport and localization	Intracellular cholesterol transport
AAEL023454	Mitochondrial carrier protein	−3.4692	0.0159	Transport and localization	Mitochondrial transporter with putative role in respiration
AAEL019798	Venom allergen	−1.7963	5.00E−18	Unknown	Detoxification, putative

a CNS, central nervous system; DEG, differentially expressed gene; JAK/STAT, Janus kinase/signal transducer and activator of transcription; IMD, immune deficiency.

Of the 32 genes that experienced significantly decreased expression as a result of biopesticide exposure in Pati mosquitoes, 10 were noncoding RNAs. Three genes were involved in small-molecule transport for protons (AAEL022056), proteins (AAEL022660), and fatty acids (AAEL009561). There were three putative immune genes, two endonucleases (AAEL022558 and AAEL027718) and *pias2*. Three genes fell into the “response to stimulus” category: a lethal essential-for-life protein with a heat shock protein 20 domain (AAEL013352), an uncategorized cytochrome p450 (AAEL000340), and an odorant binding D7 protein (AAEL006424).

A total of 145 genes showed an increased expression in Sing selected mosquitoes, but none of these genes was strongly linked to host response to stimulus, xenobiotic metabolism, or detoxification. Twenty-one of these genes were noncoding RNAs. A further 27 were zinc finger proteins, primarily from the C2H2 superfamily, with these genes likely involved in nucleic acid binding. Four genes had immune functions, including *pias2*, and three genes were putatively involved in bacterial recognition (AAEL011001, AAEL019903, and AAEL025170). A total of 10 DEGs had roles in development, including central nervous system development/growth (AAEL004130, AAEL007667, and AAEL013544), eggshell development (AAEL022628), wing/abdomen development (AAEL025990), and segment localization (AAEL012107). Four additional genes were linked to reproduction, and all four were involved in vitellogenesis and follicle development (AAEL006138, AAEL006563, AAEL018103, and AAEL021453).

In Sing mosquitoes, we observed lower expression of nine genes involved in response to stimulus. Five of these genes are likely involved in detoxification or xenobiotic metabolism. This list includes an insect allergen-like protein (AAEL009166), carboxy/choline esterase alpha esterase CCEAE1A (AAEL005113), cytochrome P450 CYP301A1 (AAEL014594), glutathione *S*-transferase E4 (AAEL007962), and a carboxylic ester hydrolase (AAEL008532). Conventionally, genes with this function would be expected to be upregulated in insecticide-resistant mosquitoes (39). A further gene in this category was putatively involved in cyanide detoxification (AAEL005759). We also observed a lower expression of 13 genes involved in protein digestion via trypsin activity. Five genes were linked to development, with three being hemolymph juvenile hormone-binding proteins (AAEL001323, AAEL004987, and AAEL026278). There were three immune genes affected, a Toll-like receptor protein (AAEL021586) and two Gram-negative binding proteins (AAEL000652 and AAEL009178). Finally, we observed that 50 genes in this category were involved in metabolism or transport, with multiple genes linked to chitin metabolism or the metabolism and transport of lipids, sugars, and other small molecules.

To understand the base differences in gene expression between the Pati and Sing colonies, we compared the transcriptomic profiles of the two sibling lines. We found 528 DEGs (Data Set S5), with 272 linked to the Pati line and 256 linked to the Sing line. The functional breakdown of these genes was highly similar between the two lines (Fig. 6c), suggesting that two colonies might utilize slightly different gene networks to complete essential biological processes. For instance, there were 27 differentially expressed proteases (Pati −17, Sing −10), potentially suggesting different approaches to proteolysis. Another major difference between the two lines was the expression of detoxification genes. We observed that there were 3 differentially expressed cytochromes p450 associated with the Pati sibling line and 14 with the Sing line, while 3 glutathione *S*-transferases also showed increased expression in the Sing colony. These findings indicate that there are potentially innate differences in detoxification activity between the two colonies.

10.1128/mbio.00480-23.5 DATA SET S5 DEGs (Pati sibling vs Sing sibling). Download Data Set S5, XLSX file, 0.06 MB. Copyright © 2023 Engdahl et al. 2023 Engdahl et al. https://creativecommons.org/licenses/by/4.0/ This content is distributed under the terms of the Creative Commons Attribution 4.0 International license.

The Pati sibling line had higher expression levels of five genes involved in mitochondrial function and the electron transport chain, plus a further two genes linked to ATP synthesis, with these results being suggestive of differences in cellular energy profiles. Chitin molecular processes were another key point of difference, with 15 genes in this functional category differentially expressed between the two lines, including peritrophins, cuticle proteins, and chitin-binding proteins. There were few differences in developmental genes; however, the Pati line showed a higher expression of AAEL026383, which is an ortholog of the Drosophila melanogaster prothoracicotropic hormone gene that regulates ecdysone production and contributes to the larval-to-pupal transition. Pati sibling mosquitoes also exhibited higher expression of hemolymph juvenile hormone-binding protein (AAEL000500), with this class of gene known to protect juvenile hormone from hydrolysis. In terms of immunity, the two lines differed mostly in bacterial recognition. The Pati sibling line had six immune DEGs, including two c-type lectins (AAEL000563 and AAEL005482), two Gram-negative binding proteins (AAEL001882 and AAEL009178), an autophagy protein (AAEL021061), and a putative regulator of the JAK-STAT pathway (AAEL025786). On the other hand, the Sing sibling line had higher expression levels of a c-type lysozyme (AAEL003723) and a glycine-rich repeat antimicrobial peptide (AAEL017536).

### Exposure to commonly used insecticides.

**FIG 7:**
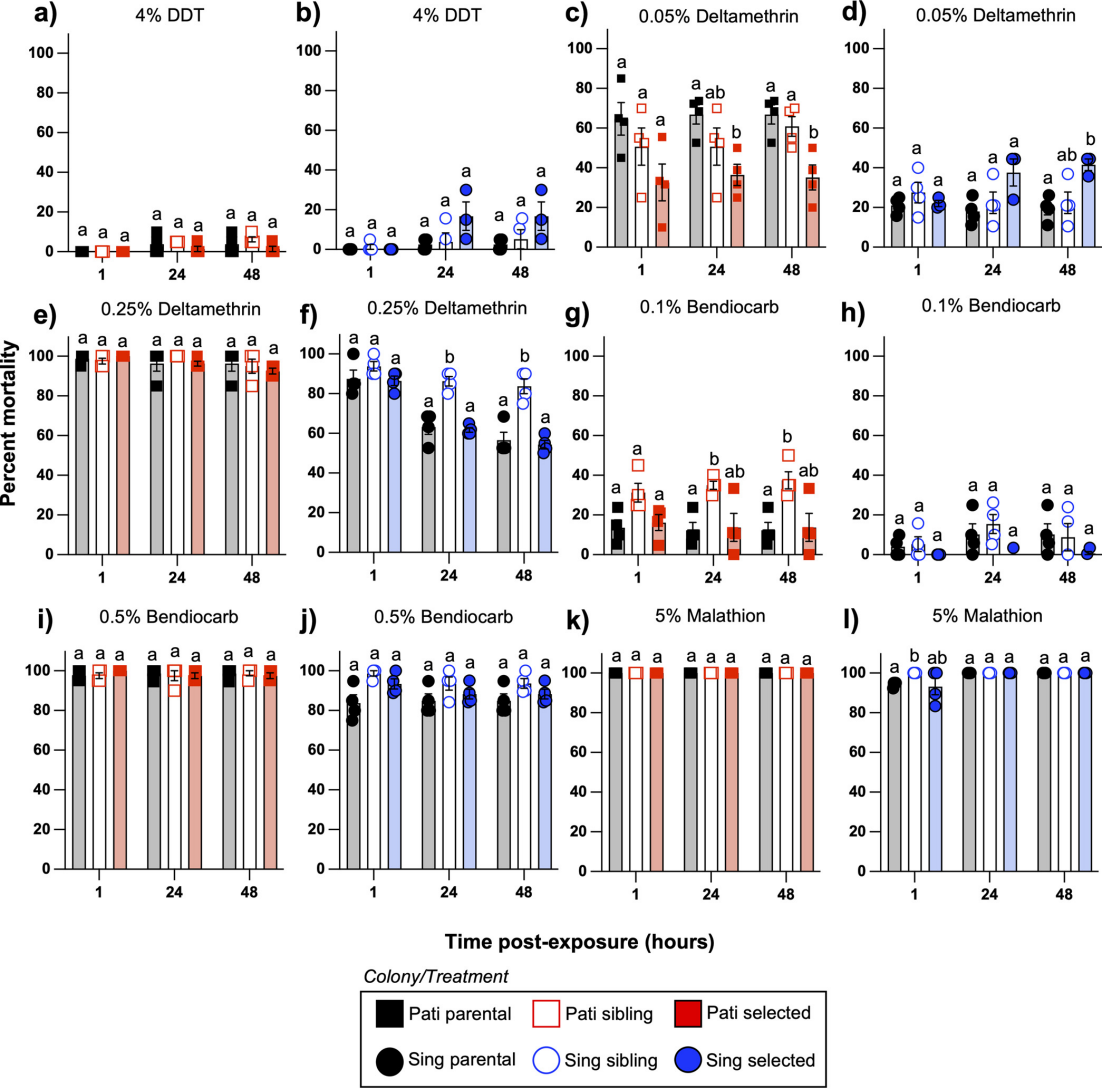
Aedes aegypti mosquitoes exposed to the Csp_P biopesticide remain susceptible to common chemical insecticides. Adult female mosquitoes from the parental, sibling, and selected lines from the Pati and Sing colonies were exposed to four different chemical insecticides using the WHO adult insecticide susceptibility test protocol. The tested compounds and concentrations were 4% dichlorodiphenyltrichloroethane (DDT) (a, b), 0.05% deltamethrin (c, d), 0.25% deltamethrin (e, f), 0.1% bendiocarb (g, h), 0.5% bendiocarb (i, j), and 5% malathion (k, l). We observed that selected mosquitoes maintained a level of susceptibility equal to or greater than that of their parental lines for all regimens, with the exception of 0.05% deltamethrin. In that assay, Pati selected mosquitoes were more resistant than Pati parental or sibling lines, but a similar effect was not observed for the Sing colony, and no resistance to deltamethrin was detected in the Pati selected line at the higher dosage of 0.25%. These observations suggest that multigenerational exposure to the Csp_P biopesticide does not consistently induce resistance to deltamethrin. In the figure, each data point represents the percentage survival value of a tube of approximately 20 adult female mosquitoes. Bar graphs depict mean values of three or four experimental replicates, and the bars represent the standard error of the mean. The letters above the data represent different statistical groups for each set of three mosquito lines, as determined by Tukey’s *post hoc* tests.

To assess whether the multigenerational exposure to the Csp_P biopesticide altered the susceptibility of Ae. aegypti to common chemical insecticides, we exposed mosquitoes from the parental, sibling, and selected lines of each colony to representative compounds of four common chemical insecticidal classes. Susceptibility to DDT (organochlorine), deltamethrin (pyrethroid), bendiocarb (carbamate), and malathion (organophosphate) was evaluated using the WHO susceptibility test for adult mosquitoes (Data Set S6). For 4% DDT, we observed no significant impact of biopesticide exposure on mortality for either the Pati (Fig. 7a; two-way ANOVA; Line – *P = *0.2402) or Sing colonies (Fig. 7b; two-way ANOVA; Line – *P = *0.1452). For 0.05% deltamethrin, we observed significantly lower mortality for Pati selected mosquitoes at 24 and 48 h postexposure (Fig. 7c; two-way ANOVA; Line – *P = *0.0291), with a mean mortality approximately half that seen in the parental line. No significant differences in survival were observed between the Pati parental and sibling lines for that treatment. In contrast, for Sing colony mosquitoes exposed to 0.05% deltamethrin, there was no significant drop in mortality associated with selection (Fig. 7d; two-way ANOVA; Line – *P = *0.1170), and the selected line actually experienced significantly higher mortality than that of the parental line at 48 h postexposure (Tukey’s test; *P = *0.0084). For 0.25% deltamethrin, we observed no significant differences between any of the three lines in the Pati colony (Fig. 7e; two-way ANOVA; Line – *P = *0.8900). However, line was a significant factor for the Sing colony (Fig. 7f; two-way ANOVA; Line – *P < *0.0001), with this effect reflecting the higher mortality in the sibling line at 24 and 48 h postexposure.

10.1128/mbio.00480-23.6 DATA SET S6 Insecticide resistance assay data. Download Data Set S6, XLSX file, 0.02 MB. Copyright © 2023 Engdahl et al. 2023 Engdahl et al. https://creativecommons.org/licenses/by/4.0/ This content is distributed under the terms of the Creative Commons Attribution 4.0 International license.

For 0.1% bendiocarb, Pati sibling mosquitoes experienced higher mortality than the other two lines (Fig. 7g; two-way ANOVA; Line – *P = *0.0139), but there was no significant effect of line for the Sing colony (Fig. 7h; two-way ANOVA; Line – *P = *0.1566). For 0.5% bendiocarb, no significant effects due to mosquito line were observed for either the Pati (Fig. 7i; Two-way ANOVA; Line – *P = *0.9646) or Sing colonies (Fig. 7j; two-way ANOVA; Line – *P = *0.0679). For the 5% malathion assay, 100% mortality was achieved within 1 h for all the Pati mosquitoes, rendering statistical comparison of the data irrelevant (Fig. 7k). Similarly, for the Sing colony, 100% mortality was reached for all mosquitoes at 24 h after exposure, and no significant effect of line was observed (Fig. 7l; two-way ANOVA; Line – *P = *0.1576).

### DENV infection.

**FIG 8:**
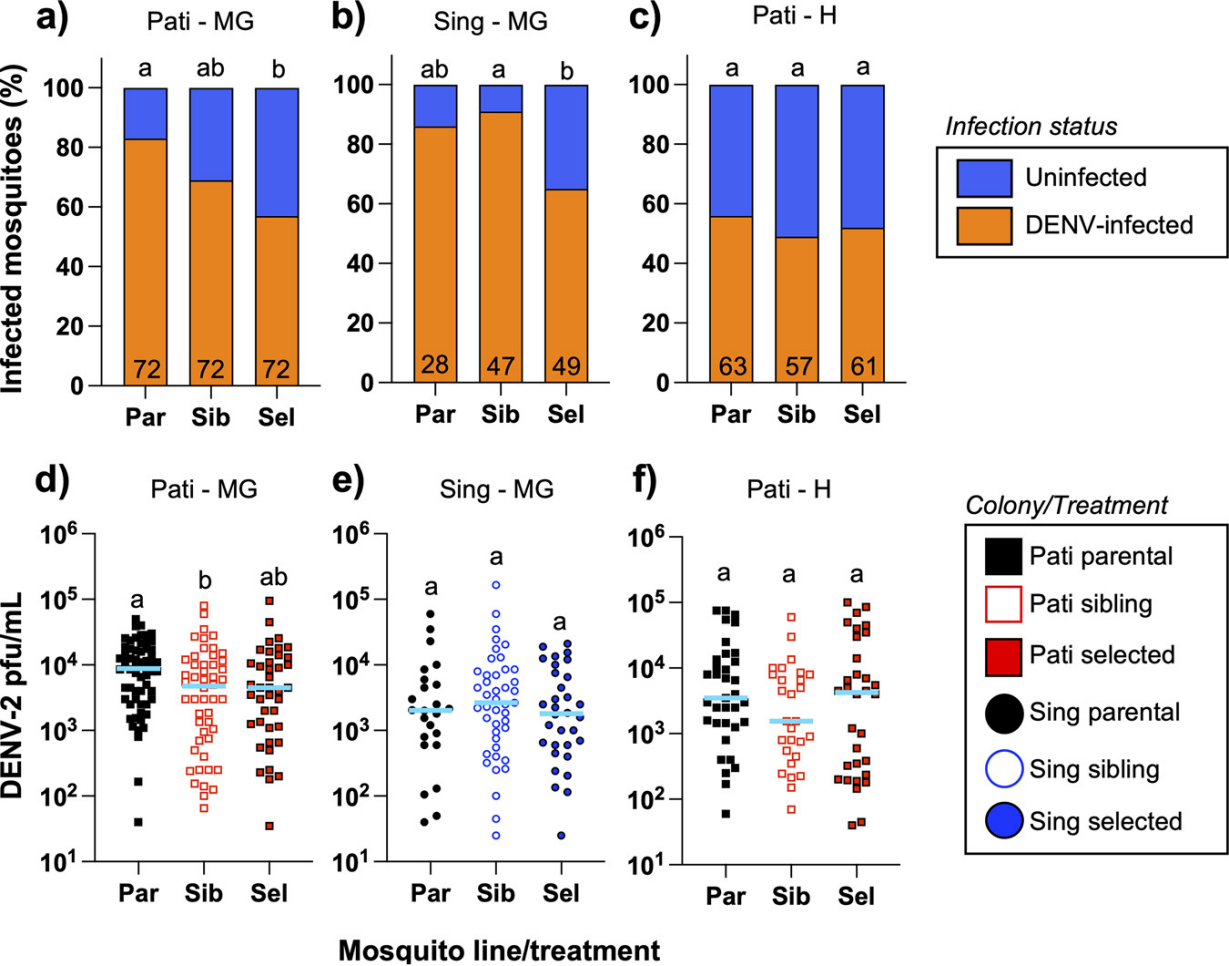
Csp_P biopesticide-exposed Aedes aegypti show evidence of decreased susceptibility to dengue virus 2 (DENV-2) infection, but DENV-2 load is unaffected. Adult females from the parental, sibling, and selected lines from the Pati and Sing colonies were orally challenged with the DENV-2 NGC strain. (a) At 7 dpi, we observed that the prevalence of infection in Pati midguts was significantly lower for sibling and selected mosquitoes than for parental mosquitoes. (b) Similarly, for Sing midguts, selected mosquitoes had a lower prevalence of infection than the sibling line. (c) However, at 14 dpi, no differences in the prevalence of DENV-2 infection were observed in Pati head samples. (d) When we compared the DENV-2 load in infected samples, Pati midguts at 7 dpi from the sibling line had significantly fewer plaque-forming units than did the parental line. (e, f) We observed no significant differences between mosquito lines for Sing midguts at 7 dpi (e) or Pati heads at 14 dpi (f). The letters above the data sets indicate statistical groups as determined by Fisher’s exact test (infection prevalence) or Dunn’s test (DENV-2 load). For prevalence panels, the numbers at the base of each bar represent the overall sample size. For viral load panels, each data point represents a measurement from a single mosquito. Solid blue lines indicate treatment medians. The data are pooled from three independent replicates. H, head; MG, midgut; Par – parental line; PFU/mL, plaque-forming units per mL of sample homogenate; Sel, selected line; Sib, sibling line.

The impact of multigenerational exposure to the Csp_P biopesticide on DENV infection was assessed in the Pati and Sing parental, sibling, and selected lines via plaque-forming assays (Data Set S7). For midguts collected at 7 days post-infection (dpi), we observed significant differences in the prevalence of infection for both Pati (Fig. 8a; chi-square test; χ^2^ = 16.03, *df *= 2, *P = *0.0003) and Sing (Fig. 8b; chi-square test; χ^2^ = 24.41, *df *= 2, *P < *0.0001) mosquitoes. These effects were due to a significantly lower infection prevalence in the selected line compared to either the sibling or parental lines. No difference in prevalence of infection was observed for Pati head samples collected at 14 dpi (Fig. 8c; chi-squared test; χ^2^ = 0.9888, *df *= 2, *P = *0.6099).

10.1128/mbio.00480-23.7 DATA SET S7 DENV infection assay data. Download Data Set S7, XLSX file, 0.01 MB. Copyright © 2023 Engdahl et al. 2023 Engdahl et al. https://creativecommons.org/licenses/by/4.0/ This content is distributed under the terms of the Creative Commons Attribution 4.0 International license.

DENV load was then compared among infected mosquitoes. For Pati midguts at 7 dpi, we observed a significant effect of treatment (Fig. 8d; Kruskal-Wallis ANOVA; *W *= 7.154, *P = *0.0280); however, this effect was between the parental and sibling lines (Dunn’s test; *P = *0.0465), not the selected line. No significant differences in viral load were observed for Sing midguts at 7 dpi (Fig. 8e; Kruskal-Wallis ANOVA; *W *= 0.8945, *P = *0.6394) or Pati heads at 14 dpi (Fig. 8f; Kruskal-Wallis ANOVA; *W *= 2.005, *P = *0.3670). Multiple linear regression of viral load across all the lines and treatments revealed no significant differences that could be attributed to mosquito line, treatment, tissue, or any interaction effect.

## DISCUSSION

In this study, we exposed juvenile mosquitoes from two different Ae. aegypti colonies to a sublethal dose of a Chromobacterium Csp_P-based biopesticide for nine consecutive generations to see whether they would develop resistance. Our results reveal three key insights into the nature of the biopesticide’s impacts on mosquito biology. First, we saw no evidence of resistance to the Csp_P biopesticide at the physiological level. Second, we saw no evidence of increased susceptibility to DENV-2 infection postexposure, and we observed a decreased susceptibility to a chemical insecticide associated with Csp_P biopesticide exposure in only 1 of 12 assays. Third, we observed no clear evidence of resistance at the molecular level, given that genes involved in xenobiotic metabolism and detoxification were not upregulated in response to biopesticide exposure. Collectively, these findings provide little indication that resistance had developed over the course of selection, suggesting that the Csp_P biopesticide is a promising tool for use in the control of mosquito populations.

### Physiological impact of multigenerational exposure to the Csp_P biopesticide.

Over the course of the selection regimen, our data strongly indicate that resistance does not arise in mosquitoes exposed to a dosage of this biopesticide that is intended to kill approximately 50% of larvae. Similarly, resistance to B. thuringiensis subsp. *israelensis* did not develop in Ae. aegypti after multigenerational exposure to B. thuringiensis subsp. *israelensis* (41). These results are in contrast to other studies that have observed resistance in mosquitoes after multigenerational exposure to insecticides (34, 35, 39) or in Drosophila after multigenerational exposure to bacteria (42). We monitored mortality over the nine generations of selection by daily counting of eclosed adult mosquitoes. In each generation, we observed significantly higher mortality associated with the biopesticide-exposed selected lines than in their counterpart sibling lines, which were bottlenecked but not exposed. Critically, we did not observe a trend toward increased survival rates over the course of the experiment, indicating that neither selected line had become more resistant to the biopesticide over time. We consistently observed a delay in the development time of about 5 to 7 days associated with biopesticide exposure over the entire selection regimen. We had expected that this developmental delay would decrease or disappear if resistance were developing. In fact, in the final generations of the experiment, the mean time to pupation and eclosion were increasing for both selected lines. As a parallel to these metrics, the resistance ratios for the Pati mosquitoes were trending sharply downward, indicating that both the selected and sibling lines were becoming more susceptible to the Csp_P biopesticide over time, whereas the ratios for the two Sing lines were flat, indicating that no change in susceptibility had occurred. Although it is difficult to demonstrate a negative, each of these observations refutes the notion that resistance to the biopesticide arose during our experiment.

Postselection fitness experiments indicated that multigenerational Csp_P biopesticide exposure consistently led to decreased longevity, with this effect being observed in all assays. The mosquitoes used in these experiments were the offspring of exposed mosquitoes but were not exposed themselves. The scale of this longevity decrease varied from 1 day in blood-fed Sing females to 15 days for blood-fed Pati females, based on median longevity times. Given the role of longevity in vectorial capacity, if similar decreases were observed after Csp_P biopesticide exposure in nature, any exposed mosquito larvae that survived to adulthood would reasonably be expected to be less likely transmitters of medically important pathogens. Fecundity, fertility, and wing length assays did not reveal other consistent fitness costs attributable to biopesticide exposure between the two mosquito colonies. The only other fitness effects observed were decreased fecundity for the Sing selected line and decreased wing size for the Pati selected line. The fact that these effects were observed only in one colony and not the other might indicate that beyond the longevity decrease, there is no predictable or universal pattern of fitness effects associated with multigenerational Csp_P biopesticide exposure. To our knowledge, few insecticide/selection studies have assayed fitness traits after selection, with one study indicating that multigenerational B. thuringiensis subsp. *israelensis* exposure had no impact on development, feeding, or reproduction traits in Ae. aegypti (38).

### Impact of multigenerational Csp_P biopesticide exposure on mosquito control and vector competence parameters.

Our postselection experiments revealed that multigenerational exposure to the Csp_P biopesticide is unlikely to have a negative impact on insecticide resistance or DENV transmission. We observed high levels of susceptibility to the four different insecticides we examined, with little consistent evidence that exposure to the Csp_P biopesticide decreased susceptibility to those chemicals. In fact, we observed a lower mortality, postexposure, associated with selection in only 1 of 12 assays, for 0.05% deltamethrin in the Pati colony. Importantly, a similar effect was not observed in the parallel assay with the Sing colony, suggesting that these results were unlikely to be indicative of a consistent response across mosquito genotypes. In the other 11 assays, we observed that the selected line had equivalent susceptibility or greater susceptibility to chemical insecticides than the parental line. Similarly, Ae. aegypti selected for resistance against B. thuringiensis subsp. *israelensis* do not display decreased susceptibility to temephos or diflubenzuron (41). Our findings suggest that there is great potential for using the Csp_P biopesticide synergistically with a variety of chemical insecticides in an integrated pest-management strategy.

The results from our DENV oral infection assays demonstrate that exposure to the Csp_P biopesticide does not increase susceptibility to DENV-2 infection. We did observe several significant effects of viral infection associated with selection. However, they were all decreases in prevalence or viral load associated with selection. The effects were observed in both the Pati and Sing mosquito midguts collected at 7 dpi but not in the Pati heads collected at 14 dpi. As each of these significant effects occurred between the selected line and only one of the sibling or parental lines, it is difficult to suggest that exposure to the Csp_P decreased susceptibility to DENV infection. If such an effect proved to be repeatable, it would likely translate to a minor delay in the viral replication cycle or an extension of the extrinsic incubation period. Coupled with the decrease in longevity, sublethal exposure to the Csp_P biopesticide could feasibly lead to significant perturbations of typical vectorial capacity for DENV-2. Consequently, this situation merits further investigation with other DENV isolates or other arboviruses.

Other studies have investigated links between resistance and vector competence. Resistant Culex quinquefasciatus mosquitoes experience increased rates of West Nile virus dissemination (43), and glutathione *S*-transferase-mediated resistance in Anopheles funestus increases susceptibility to P. falciparum infection (44). In A. gambiae, resistance modulates mosquito-*Plasmodium* interactions, with resistant mosquitoes being more susceptible to P. falciparum infection and with one *kdr* mutation also being linked to lower oocyst load (45). Finally, in a similar selection experiment involving Ae. aegypti and B. thuringiensis subsp. *israelensis*, selected mosquitoes experienced a higher rate of disseminated ZIKV infection (38). Exposure to live Csp_P has an antiviral effect in Ae. aegypti (27, 29). Given that the biopesticide does not contain live bacterial cells that would produce the antiviral protease and that the biopesticide is air-dried, we consider it unlikely that the protease is present in high abundance or even viable in the biopesticide preparation. Thus, potential antiviral activity by the nonlive Csp_P biopesticide may be mediated through a different mechanism.

### RNA-Seq reveals no conclusive molecular evidence for resistance against the Csp_P biopesticide.

Our RNA-Seq-based transcriptomic profiling of selected line mosquitoes from both the Pati and Sing colonies revealed there was no consistent transcriptional response to Csp_P exposure between the colonies and that there was little evidence of increased potential to detoxify or metabolize the biopesticide, with expression changes in these gene families being linked to resistance to insecticides such as imidacloprid, propoxur, and permethrin (35, 39, 46). Our data indicate that the molecular response to Csp_P biopesticide exposure of the two mosquito colonies differs in scale and scope, with a greater number of functionally diverse DEGs associated with the Sing colony. A key finding in the comparison of the DEGs between the two colonies is that only five DEGs were shared, and four of those five genes were noncoding RNAs. These observations suggest that there was no common transcriptional response to Csp_P biopesticide exposure.

When we compared the DEGs associated with selection to transcriptomes from other insecticide-resistant mosquitoes (35, 39, 40, 46), we saw little evidence for molecular resistance developing against the Csp_P biopesticide. Metabolic resistance to insecticides is typically mediated by glutathione *S*-transferases, α/β-esterases, and oxidases, particularly cytochrome p450, that fit into the “response to stimulus”’ functional category in our data. We see little evidence that genes in these classes are upregulated after exposure to the Csp_P biopesticide. In fact, for Pati mosquitoes, the only three “response to stimulus” category DEGs showed decreased expression after selection, while for Sing mosquitoes, there were nine genes in that category with decreased expression. These 12 genes include two cytochromes p450, a carboxylic ester hydrolase, carboxy/choline esterase α-esterase (*CCEAE1A*), an insect allergen-like protein, and glutathione *S*-transferase E4 (*GSTE4*). All 12 have a role in detoxification, suggesting that the Sing selected line actually exhibited decreased detoxification potential compared to its sibling counterpart.

Penetration resistance in insects is typically associated with a thickening of the cuticle, which decreases the ability of the insecticides to penetrate the cuticle (35, 39, 40, 46). If increased penetrative resistance were to have resulted from our selection regimen, we might expect to see an increase in chitin biosynthesis and potentially an increased expression of chitin-binding and cuticular proteins. For Pati mosquitoes, we observed no DEGs that fell into that functional category. For Sing mosquitoes, we identified 14 DEGs with a structural role: 8 of them were cuticular proteins, and 2 were chitin-binding proteins, but all 14 DEGs showed decreased expression in response to selection. Given that the delivery method for the Csp_P biopesticide in our study was oral rather than topical, it is unclear whether typical mechanisms for penetrative resistance would facilitate resistance here, but in any case, our data suggest that this type of resistance did not occur in our experiment.

### Study caveats.

Issues of yield made it necessary to use multiple batches of the Csp_P biopesticide in the selection regimen. Because these batches differed in their larvicidal activity, we were obliged to adjust the dose provided to the mosquito larvae accordingly, which meant that we were unable to provide a standardized selection regimen across all the generations. Consequently, fluctuations in mortality and development time did occur, but these fluctuations were in line with variation in the dose of biopesticide. This batch-to-batch variation was likely a product of a benchtop-based culturing protocol and would be expected to be eliminated if the biopesticide were to be cultured on a larger scale in a bioreactor. Another note about our study design is that the bottlenecking of the sibling lines took place at the larval stage rather than the blood feeding stage; this occurred because of the developmental delay associated with selection, which meant that the lines were blood-fed at different times. For several of the postselection fitness assays, issues of low egg availability (particularly for the Sing colony) unfortunately limited the availability of sibling line mosquitoes; the availability of those lines would have produced a more comprehensive data set. Our data demonstrate no clear or significant evidence that resistance to the Csp_P biopesticide developed. However, we cannot conclusively state that resistance would never arise. We performed our assay over nine or ten generations of exposure to the Csp_P biopesticide, which was a sufficient time to detect resistance in other studies (35, 39). It is possible that resistance could have emerged if we had continued the assay over further generations, as other studies have involved a selection for periods of approximately 30 generations (38). It is also possible that resistance to the biopesticide could emerge more readily in the field, under various environmental conditions, or in other populations of Ae. aegypti or other mosquito species.

### Conclusions and future directions.

Our data indicate that a suboptimal treatment regimen with the Csp_P biopesticide that kills approximately half of the mosquitoes that are exposed is very unlikely to lead to the development of resistance. Moreover, the descendants of the mosquitoes that survive experienced decreased longevity, remained highly susceptible to other insecticide classes, and showed evidence of slightly decreased susceptibility to DENV-2. All of these findings highlight the Csp_P biopesticide as an exciting, emerging mosquito control tool. Future research in this field will likely focus on the impact of other formulations of the biopesticide since adjustments to culture methods and delivery systems are currently ongoing. Once these adjustments are made, it will be very interesting to examine the physiological and molecular effects of sublethal exposure to the Csp_P biopesticide in mosquito populations under field conditions.

## MATERIALS AND METHODS

### Mosquito rearing.

Our experiments involved two different colonies of Ae. aegypti, each isolated from distinct geographic locations where DENV transmission is endemic. The first colony, termed Sing, was isolated from field-collected mosquitoes in Singapore in 2010 (47). The second, Pati, was originally isolated from Patillas, Puerto Rico, in 2012 and then maintained by the CDC Dengue Branch (48) before being transferred to Johns Hopkins University in 2019. All colony mosquitoes were maintained in a climate-controlled insectary with an ambient temperature of 27°C, a relative humidity of 80%, and a 12-h light:dark cycle. Colony larvae and larvae used in the fitness assays detailed below were maintained at a low density of 100 larvae/liter of deionized water to limit competition effects and were fed on TetraMin tropical tablets. Colony adults were maintained in 25 × 25 × 25-cm cages and fed on 10% sucrose solution, changed twice weekly. To produce eggs for mosquito colonies and during experiments, the mosquitoes were allowed to feed on anesthetized Swiss Webster mice (Charles River Laboratories, Rockville, MD). The use of mice for blood feeding in these experiments was approved by the Johns Hopkins University Animal Care and Use Committee (permits RA21H388 and MO18H82). During the experiments, small cohorts of adult mosquitoes were maintained in paper cups (volume of 237 or 473 mL for the longevity assays) covered by mesh netting.

### Biopesticide preparation and delivery.

Csp_P biopesticide powder was prepared as previously described (32). Note that in that article, the preparation we used is referred to as “nonlive_1.” In brief, live Csp_P from a frozen stock were inoculated in Luria-Bertani (LB) broth and grown to log phase. These cultures were mixed 1:1 with 50% glycerol in water, spread on sterile 400-cm^2^ LB agar plates, and then incubated at 30°C for 48 h. LB broth was then added to the plates to support biofilm formation, and the plates were incubated at room temperature for 5 additional days. The broth was then decanted, and the biofilm was scraped off the plates, air dried, and then ground into a fine powder. The Csp_P powder was stored at room temperature and then incorporated into baits, which consisted of 2 mL of 20% agar and 400 mg of fishmeal (Dirty Gardener).

### Selection regimen and measurement of developmental parameters.

Pati and Sing larvae were exposed to the Csp_P biopesticide for nine generations (G_1_ through G_9_), during which time developmental parameters were monitored daily. To generate sufficient numbers of eggs for fitness assays, insecticide susceptibility assays, and virus infection assays, a tenth generation of selection (G_10_) was run under the same conditions, but without monitoring of development parameters. Prior to biopesticide exposure, colony larvae were split into two cohorts. Larvae from the selected cohort were exposed to a dose of the Csp_P biopesticide provided in a food pellet, with a dosage intended to kill approximately 50% to 70% of larvae before pupation. To achieve that level of mortality and to account for variation in activity between batches of the biopesticide, we varied the dose between generations. Dosage and biopesticide batch details were as follows: G_1_ to G_3_ were batch 1, with 70 mg/pellet for G_1_, 90 mg/pellet for G_2_, and 150 mg/pellet for G_3_; G_4_ was batch 2, with 100 mg/pellet; and G_5_ to G_10_ were batch 3, all with 130 mg/pellet. Batches of the biopesticide were prepared under identical conditions and from the same bacterial stock culture but at different times.

Larvae from the sibling cohort were given a control pellet that did not contain any biopesticide. In each generation, eggs from the Pati and Sing selected and sibling lines were hatched independently, but the sibling lines were bottlenecked to 1,200 larvae to mimic the reduction in population size experienced by the selected lines. At 3 days after hatching, groups of 300 larvae from each cohort were transferred to trays containing 3 liters of deionized water. A total of four trays from each sibling line and six trays from each selected line were maintained in each generation, with the higher number of selected line trays required to produce sufficient adults to seed subsequent generations, given the higher death rate they experienced. New food pellets were added to trays every 3 days.

In each generation, three developmental parameters were monitored daily for each tray: the number of new pupae, the number of dead pupae, and the number of newly eclosed adults. New pupae were transferred to plastic cups containing deionized water and allowed to emerge freely into cages. Dead pupae were removed from trays and cups. The numbers of newly eclosed adults from each tray were measured via pupal exuviae, which were counted and then removed from pupal cups. Adults from each treatment were allowed to emerge into a single cage. Adults were blood-fed, as described above, and eggs were dried down and used for subsequent generations of selection. Parental lines for the Pati and Sing colonies were maintained without selection pressure and reared and maintained as described above. Large stocks of early-generation parental eggs were generated, and G_1_ and G_2_ parental eggs were used in subsequent experiments, while G_3_ eggs were used for some postselection assays.

### Calculation of LD_50_ and resistance ratios.

During G_5_ to G_8_ and for G_10_ of the selection regimen, tolerance to the Csp_P biopesticide was evaluated by exposing first instar selected, sibling, and parental larvae from the Pati and Sing colonies to food pellets containing 0, 5, 10, 50, 100, or 200 mg of the biopesticide. Larval survival was then monitored for 7 days. Each dose was repeated for 3 independent cohorts of 10 larvae in the wells of a 6-well plate, and the LD_50_ was estimated based on average survival values across treatments. Resistance ratios were calculated by comparing the LD_50_ of selected or sibling lines to that of their parental line. A resistance ratio of greater than 1 indicated that the line was more tolerant of the Csp_P biopesticide than the parental colony, and an increase in the ratio across generations indicated that a line had become more tolerant of the biopesticide during the selection regimen. Because of the low numbers of eggs in the Pati sibling line during G_8_, LD_50_ and resistance ratio data were not collected for this generation. Similarly, these data were not collected during G_9_ because the majority of the G_9_ eggs for all lines were used in the fitness, insecticide susceptibility, and DENV infection assays.

### Impact of biopesticide exposure on mosquito fitness.

At G_9_/G_10_, we investigated the impact of multigenerational exposure to the Csp_P biopesticide by examining four life history traits: longevity, fecundity, fertility, and body size. No larvae involved in these assays were exposed to the Csp_P biopesticide. Instead, all larvae were fed on TetraMin tropical tablets.

For the fecundity and fertility assays, mosquitoes in 25 × 25 × 25-cm cages, each containing adult male and female mosquitoes from one of the treatment groups, were allowed to mate freely. At 5 days posteclosion, the mosquitoes were given the chance to feed on anesthetized mice. Fully engorged females were separated from males and unfed females. Three days after feeding, the females were knocked down and transferred to individual fly vials (h: 95 mm, ø: 28.5 mm) containing a damp cone of filter paper for oviposition. Egg papers were removed 6 days after feeding, then photographed, and manually counted in photo viewing software (Microsoft Paint). Vials in which the female did not survive until 6 days postfeeding were discarded. Fertility assays were conducted with the eggs from the fecundity assays described above. Oviposition papers were dried individually in petri dishes, and once dried, they were submerged in hatching water (one ground TetraMin tablet dissolved in 1 liter of deionized water and then autoclaved). Three days later, the total number of live larvae per paper was counted manually, and the hatching rate was calculated as a percentage of total eggs laid. Wing length was calculated as a proxy for adult body size. Adult mosquitoes were anesthetized at 4°C, and their wings were measured from the axial vein near the thorax to the tip of the R1 vein under a microscope using a 0.1-mm stage micrometer.

The longevity of sugar-fed females (virgin, non-blood-fed), sugar-fed males (virgin), and blood-fed females (virgin) was compared between the selected and parental lines from the Pati and Sing colonies. To generate virgin adults, male and female pupae were manually separated into different cages. There were insufficient mosquitoes from either sibling line to include those lines in this assay. Within 24 h posteclosion, cohorts of 20 to 30 adult mosquitoes were transferred to paper cups. Additional cups of females were blood-fed on mice at 5 days posteclosion. After feeding, cohorts of approximately 30 fully engorged females were transferred to new cups. Cups were supplied with a sterile 10% sucrose solution on cotton, which was changed every second day. Dead mosquitoes were counted and removed from each cup every 1 to 2 days until all the mosquitoes had died. The cups were changed every 1 to 3 weeks to minimize mosquito exposure to microorganisms that could grow in sucrose droplets or mosquito excreta.

### Transcriptomic impact of exposure to the Csp_P biopesticide.

To examine the molecular impact of multigenerational exposure to the Csp_P biopesticide, we used RNA-Seq to profile the transcriptomes of Pati and Sing mosquitoes from the G_10_ selected and sibling lines compared to the G_1_ parental lines. At 5 to 7 days posteclosion, 3 pools of 10 mosquitoes were collected from each of the 6 lines. Each pool consisted of five female and five male mosquitoes, with a mix of sexes used to minimize the presence of sex-specific transcriptional effects in the final data. RNA from the mosquito pools was extracted using a RNeasy minikit (Qiagen, catalog no. 74104) according to the manufacturer’s instructions. In brief, mosquito pools were homogenized in 200 μL of RLT buffer and β-mercaptoethanol using a handheld electric homogenizer and a micropestle. RNA quality was assayed at the Genomic Analysis and Sequencing Core Facility at Johns Hopkins University. Sample RNA integrity numbers (RINs) were determined using a TapeStation (Agilent). Samples with RIN values above 9 were approved for analysis. Samples were then sent to Novogene (Sacramento, CA, USA), who performed the library preparation, mRNA sequencing using the Illumina NovaSeq 6000 platform, and then the initial bioinformatic analysis, including mapping to the Ae. aegypti reference genome (AaegL5.0; GCA_002204515.1) and calculated read counts and fragments per kilobase of transcript sequence per million base pairs sequenced (FPKM).

Differential gene expression analysis via the DESeq2 package in R (49) was used to assess significant differences in expression between treatments, considering expression data across triplicate samples from each treatment. A false discovery rate of 5% and a 2-fold change in expression were set as the thresholds for differential expression status. Comparisons between the Pati and Sing sibling lines were performed, *post hoc*, using the same approach. We were interested in identifying differentially expressed genes (DEGs) that fit one of two descriptions. First, we built lists of DEGs associated with selection by identifying genes with altered expression between selected and sibling lines but not between sibling and parental lines. Second, we identified DEGs between the Pati and Sing sibling lines in order to evaluate innate differences in transcription between the two colonies. Annotation of DEGs and likely functions were completed using annotations from VectorBase (vectorbase.org/vectorbase/app), InterPro (ebi.ac.uk/interpro/), UniProt (uniprot.org), and FlyBase (flybase.org), including that of gene orthologs. Genes were then assigned a primary and/or secondary functional class based on one of 16 Gene Ontology Biological Process terms. DEGs with incomplete annotation or without annotation were assigned to the “Unknown” class. These data were used to generate profiles of DEGs affected by exposure to the Csp_P biopesticide, including those affected in both the Pati and Sing lines, and baseline transcriptomic differences between the Pati and Sing sibling lines.

### Insecticide susceptibility tests.

Susceptibility of the six mosquito lines to four commercially available chemical insecticides, bendiocarb, DDT, deltamethrin, and malathion, was investigated using the WHO susceptibility test for adult mosquitoes (50). For these chemicals, we initially utilized the diagnostic concentrations determined by the WHO and used for monitoring insecticide resistance in wild *Anopheles* mosquitoes. For bendiocarb and deltamethrin, we repeated the assays using five times the diagnostic concentration because of the low mortality seen at the diagnostic concentration. The concentrations used were: bendiocarb, 0.1% and 0.5%; deltamethrin, 0.05% and 0.25%; DDT, 4%; and malathion, 5%. The assays were conducted according to the WHO-approved protocols (50), with modifications made to account for smaller sample sizes for some mosquito lines. Assay supplies, including insecticide-impregnated filter papers, were prepared and provided by the Vector Control Research Unit, University Sains Malaysia. Bioassays involved the progeny of G_9_ or G_10_ mosquitoes, but the larvae were not subject to selection or bottlenecking. In each bioassay, female mosquitoes at 4 days posteclosion were split into 6 tubes, with each tube containing 20 mosquitoes. Four tubes were exposed to insecticides, and the remaining two served as unexposed controls. Treatment tubes were lined with insecticide-impregnated papers, and control tubes were lined with filter paper impregnated only with the carrier solution used for that particular insecticide (bendiocarb/malathion, olive oil; deltamethrin, silicon oil; and DDT, ricella oil). During the exposure period, the tubes were placed in a climate-controlled reach-in incubator (ambient temperature of 27°C, relative humidity of 80%) without light for 1 h. Afterwards, the mosquitoes were anesthetized by chilling at 4°C, transferred to paper cups, and provided with a 10% sucrose solution. Initial mortality was recorded within 10 min of transfer to the cups (i.e., equating to approximately 1 h after the initial exposure). Mortality was recorded again at 24 and 48 h postexposure.

### DENV propagation and titration.

C6/36 cells, derived from Aedes albopictus, were used for DENV propagation. The cells were cultured at 32°C with 5% CO_2_ in Eagle’s minimal essential medium (MEM) complete medium (MEM supplemented with 10% heat-inactivated fetal bovine serum [FBS], 1% l-glutamine and nonessential amino acid, and 1% [vol/vol] penicillin-streptomycin). Experimental infections were performed using frozen aliquots of the DENV-2 New Guinea C strain in culture medium. The viral titer of these stocks was estimated at 4 × 10^7^ plaque-forming units/mL through plaque-forming assay. All aliquots used were from the same batch of virus and were stored at −80°C from the time of harvesting until their use in oral infection assays.

### Oral infection of mosquitoes with DENV.

Cohorts of 60 adult female mosquitoes at 5 to 7 days posteclosion were offered an infectious bloodmeal (containing 50% DENV in culture medium, 40% RPMI-washed human red blood cells, 10% FBS, and 1% ATP (100 mM)) via an artificial membrane feeding system. The mosquitoes were allowed to feed for 1 h and were then anesthetized in a cold room (4°C). Fully engorged females were transferred to new cups, moved to a climate-controlled reach-in incubator (ambient temperature of 27°C, relative humidity of 80%), and fed on 10% sterile sucrose solution. The sucrose was changed daily, and the mosquitoes were moved to new cups every 3 days.

Midguts were collected from Pati and Sing mosquitoes at 7 dpi to assay infection. Heads from Pati mosquitoes were collected at 14 dpi to assay disseminated infection. At these time points, the mosquitoes were anesthetized at 4°C and surface-sterilized by immersion in 75% ethanol and then washed twice in sterile water before the midguts or heads were dissected using a dissection microscope. Dissected tissues were immediately transferred to 300 μL Dulbecco’s modified Eagle’s medium (DMEM) cell culture supplemented with 2% Gibco heat-inactivated FBS, 1% l-glutamine, 1% penicillin-streptomycin, and 5 μg/mL plasminogen in a deep-well 96-well plate containing sterile glass beads. The plates were covered and stored at −80°C. A total of 2 to 24 midguts or 11 to 24 heads from each treatment were used. The experiment was repeated three times with cohorts from the same egg batches and with aliquots from the same DENV batch.

Viral infection parameters (prevalence of infection and viral load) were assessed via plaque-forming assay using baby hamster kidney (BHK21) cells. The cells were cultured for 5 days at 37°C + 5% CO_2_ in DMEM supplemented with 10% heat-inactivated FBS, 1% l-glutamine, 1% (vol/vol) penicillin-streptomycin, and plasminogen. Samples in medium were completely thawed, then homogenized in a Mini-bead-beater (Biospec Products). The homogenized samples were serially diluted in fresh DMEM (four dilutions at a 1:10 dilution factor), and then 100 μL of each dilution was added to a monolayer of BHK cells (at ~80% confluence) in 24-well plates. The plates were rocked at room temperature for 15 min and then incubated for 45 min at 37°C. Afterwards, 1 mL of prewarmed methylcellulose overlay (DMEM + 2% Gibco heat-inactivated FBS + 1% l-glutamine + 1% penicillin-streptomycin + 5 μg/mL plasminogen + 0.8% methylcellulose; autoclaved) was added per well, and the plates were incubated at 37°C with 5% CO_2_.

Six days postinfection, the cells were fixed and stained using a 1:1 acetone:MeOH + 20% crystal violet solution. Afterwards, the plaques were manually counted in the well with the lowest dilution that contained countable plaques. Prevalence was calculated as the number of virus-containing midguts per total midguts. The viral load was calculated as the number of plaques per individual mosquito, adjusted for dilution. Similarly, the dissemination intensity was calculated as the number of plaques per individual mosquito head sample.

### Statistics and reproducibility.

Statistical analysis was performed using GraphPad Prism (version 9.3.0) and R Studio (Ghost Orchid Release for macOS, 077589bc, 20 September 2021). Where appliable, the data were screened for normality using the D’Agostino-Pearson test in Prism. Non-normal data were analyzed using nonparametric statistics. The figures were prepared using GraphPad Prism and Microsoft PowerPoint (version 16.61).

**(i) Developmental assays.** Developmental assays were repeated during each of the nine generations of the selection regimen. For mean time to pupation, the analysis involved data from 8,104 to 10,393 pupae per treatment. For mean time to eclosion and survival to eclosion rates, the analyses involved data from 6,344 to 10,042 newly eclosed adults per treatment. The data for these three traits were compared using two-way ANOVA in Prism with line (selected versus sibling), generation, and line × generation interaction as the predictor variables. Statistical tests were run independently for Pati and Sing mosquitoes.

**(ii) Resistance ratios.** Resistance ratios were calculated using three cohorts of 10 larvae/dose of biopesticide for a total of 180 larvae per mosquito line. The assay was performed once each generation between G_5_ and G_10_ of the selection regimen. To analyze resistance ratio data, we plotted changes in the resistance ratios over time and then used simple linear regression in Prism to determine whether the slopes of the selected and sibling lines for each mosquito colony differed significantly.

**(iii) Fecundity and fertility and wing length assays.** Fecundity and fertility data were recorded from 20 to 62 females per mosquito line. Wing length measurements were made for cohorts of 28 to 30 female and 18 to 30 male mosquitoes per treatment. Each assay was performed once. These data were compared between treatments and the Pati and Sing colonies using multiple linear regression in Prism. *Post hoc* comparisons of data from each line were performed independently for each colony using Dunn’s multiple-comparison test.

**(iv) Longevity assay.** For the longevity assays, final sample sizes per treatment varied from 40 to 91 mosquitoes. Three replicate cups were run for each treatment group, except for the Sing blood-fed treatment, for which there were only enough adults for two cups. Each assay was performed once. Differences in longevity between treatments were compared using the Mantel-Cox test in Prism. Hazard ratios, which compare the relative risk between treatment groups, were calculated using the Mantel-Haenszel test.

**(v) Transcriptome.** Data from each treatment were generated from three independent pools of 10 adult mosquitoes (5 female and 5 male). Differential gene expression analysis was performed via the DESeq2 package in R.

**(vi) Insecticide resistance assays.** For each assay, four tubes of 20 adult female mosquitoes from the Pati and Sing selected, sibling, and parental lines were exposed to insecticide, while 2 further tubes of 20 adult females/mosquito line were left unexposed for comparative purposes. Each assay was repeated once. For data analysis, Abbott’s formula was used when the mortality in the control treatments was above 5% (51). The data for each time point were compared using two-way ANOVA in Prism followed by Tukey’s *post hoc* test, adjusted for multiple comparisons.

**(vii) DENV infection assays.** Assays of DENV prevalence in Pati mosquito midguts involved 72 guts per line. For Sing mosquito midguts, sample sizes varied from 28 to 49 guts per line. For Pati heads, sample sizes ranged from 57 to 61. Three independent feeding assays were performed, and data were combined across assays when preliminary analysis via multiple linear regression showed no significant effects due to replicate. Statistical comparisons of DENV prevalence among the three Pati or Sing lines were performed using chi-squared tests in Prism, and data were then compared pairwise using Fisher’s exact test. Viral loads were compared only for infected mosquitoes. Differences between treatments for each line by tissue combination were assessed via Kruskal-Wallis ANOVA in Prism, with *post hoc* pairwise comparisons made using Dunn’s multiple-comparison test. Viral load was compared across all treatments, tissues, and mosquito lines using a multiple linear regression model in Prism.

### Data availability.

All of the data are available in the main text or in the supplementary data. Raw RNA-Seq data have been submitted to the Sequence Read Archive (NCBI) and are available via accession number PRJNA913171.

## References

[1] Yee DA, Dean Bermond C, Reyes-Torres LJ, Fijman NS, Scavo NA, Nelsen J, Yee SH. 2022. Robust network stability of mosquitoes and human pathogens of medical importance. Parasit Vectors 15:216. doi:10.1186/s13071-022-05333-4. 35725618 PMC9208160

[2] Franklinos LHV, Jones KE, Redding DW, Abubakar I. 2019. The effect of global change on mosquito-borne disease. Lancet Infect Dis 19:e302–e312. doi:10.1016/S1473-3099(19)30161-6. 31227327

[3] Carrasco D, Lefevre T, Moiroux N, Pennetier C, Chandre F, Cohuet A. 2019. Behavioural adaptations of mosquito vectors to insecticide control. Curr Opin Insect Sci 34:48–54. doi:10.1016/j.cois.2019.03.005. 31247417

[4] Liu N. 2015. Insecticide resistance in mosquitoes: impact, mechanisms, and research directions. Annu Rev Entomol 60:537–559. doi:10.1146/annurev-ento-010814-020828. 25564745

[5] Hemingway J, Ranson H, Magill A, Kolaczinski J, Fornadel C, Gimnig J, Coetzee M, Simard F, Roch DK, Hinzoumbe CK, Pickett J, Schellenberg D, Gething P, Hoppe M, Hamon N. 2016. Averting a malaria disaster: will insecticide resistance derail malaria control? Lancet 387:1785–1788. doi:10.1016/S0140-6736(15)00417-1. 26880124 PMC6215693

[6] Caragata EP, Dong S, Dong Y, Simoes ML, Tikhe CV, Dimopoulos G. 2020. Prospects and pitfalls: next-generation tools to control mosquito-transmitted disease. Annu Rev Microbiol 74:455–475. doi:10.1146/annurev-micro-011320-025557. 32905752

[7] Caragata EP, Dutra HLC, Sucupira PHF, Ferreira AGA, Moreira LA. 2021. Wolbachia as translational science: controlling mosquito-borne pathogens. Trends Parasitol 37:1050–1067. doi:10.1016/j.pt.2021.06.007. 34303627

[8] Beebe NW, Pagendam D, Trewin BJ, Boomer A, Bradford M, Ford A, Liddington C, Bondarenco A, De Barro PJ, Gilchrist J, Paton C, Staunton KM, Johnson B, Maynard AJ, Devine GJ, Hugo LE, Rasic G, Cook H, Massaro P, Snoad N, Crawford JE, White BJ, Xi Z, Ritchie SA. 2021. Releasing incompatible males drives strong suppression across populations of wild and *Wolbachia*-carrying *Aedes aegypti* in Australia. Proc Natl Acad Sci USA 118:e2106828118. doi:10.1073/pnas.2106828118. 34607949 PMC8521666

[9] Zheng X, Zhang D, Li Y, Yang C, Wu Y, Liang X, Liang Y, Pan X, Hu L, Sun Q, Wang X, Wei Y, Zhu J, Qian W, Yan Z, Parker AG, Gilles JRL, Bourtzis K, Bouyer J, Tang M, Zheng B, Yu J, Liu J, Zhuang J, Hu Z, Zhang M, Gong JT, Hong XY, Zhang Z, Lin L, Liu Q, Hu Z, Wu Z, Baton LA, Hoffmann AA, Xi Z. 2019. Incompatible and sterile insect techniques combined eliminate mosquitoes. Nature 572:56–61. doi:10.1038/s41586-019-1407-9. 31316207

[10] Indriani C, Tantowijoyo W, Rances E, Andari B, Prabowo E, Yusdi D, Ansari MR, Wardana DS, Supriyati E, Nurhayati I, Ernesia I, Setyawan S, Fitriana I, Arguni E, Amelia Y, Ahmad RA, Jewell NP, Dufault SM, Ryan PA, Green BR, McAdam TF, O’Neill SL, Tanamas SK, Simmons CP, Anders KL, Utarini A. 2020. Reduced dengue incidence following deployments of *Wolbachia*-infected *Aedes aegypti* in Yogyakarta, Indonesia: a quasi-experimental trial using controlled interrupted time series analysis. Gates Open Res 4:50. doi:10.12688/gatesopenres.13122.1. 32803130 PMC7403856

[11] Nazni WA, Hoffmann AA, NoorAfizah A, Cheong YL, Mancini MV, Golding N, Kamarul GMR, Arif MAK, Thohir H, NurSyamimi H, ZatilAqmar MZ, NurRuqqayah M, NorSyazwani A, Faiz A, Irfan FMN, Rubaaini S, Nuradila N, Nizam NMN, Irwan SM, Endersby-Harshman NM, White VL, Ant TH, Herd CS, Hasnor AH, AbuBakar R, Hapsah DM, Khadijah K, Kamilan D, Lee SC, Paid YM, Fadzilah K, Topek O, Gill BS, Lee HL, Sinkins SP. 2019. Establishment of *Wolbachia* strain wAlbB in Malaysian populations of *Aedes aegypti* for dengue control. Curr Biol 29:4241–4248.e5. doi:10.1016/j.cub.2019.11.007. 31761702 PMC6926472

[12] Carvalho DO, McKemey AR, Garziera L, Lacroix R, Donnelly CA, Alphey L, Malavasi A, Capurro ML. 2015. Suppression of a field population of *Aedes aegypti* in Brazil by sustained release of transgenic male mosquitoes. PLoS Negl Trop Dis 9:e0003864. doi:10.1371/journal.pntd.0003864. 26135160 PMC4489809

[13] Wiltshire RM, Duman-Scheel M. 2020. Advances in oral RNAi for disease vector mosquito research and control. Curr Opin Insect Sci 40:18–23. doi:10.1016/j.cois.2020.05.002. 32516723 PMC8718359

[14] Gantz VM, Jasinskiene N, Tatarenkova O, Fazekas A, Macias VM, Bier E, James AA. 2015. Highly efficient Cas9-mediated gene drive for population modification of the malaria vector mosquito *Anopheles stephensi*. Proc Natl Acad Sci USA 112:E6736–E6743. doi:10.1073/pnas.1521077112. 26598698 PMC4679060

[15] Hammond A, Galizi R, Kyrou K, Simoni A, Siniscalchi C, Katsanos D, Gribble M, Baker D, Marois E, Russell S, Burt A, Windbichler N, Crisanti A, Nolan T. 2016. A CRISPR-Cas9 gene drive system targeting female reproduction in the malaria mosquito vector *Anopheles gambiae*. Nat Biotechnol 34:78–83. doi:10.1038/nbt.3439. 26641531 PMC4913862

[16] Berry C. 2012. The bacterium, *Lysinibacillus sphaericus*, as an insect pathogen. J Invertebr Pathol 109:1–10. doi:10.1016/j.jip.2011.11.008. 22137877

[17] Kroeger I, Liess M, Dziock F, Duquesne S. 2013. Sustainable control of mosquito larvae in the field by the combined actions of the biological insecticide Bti and natural competitors. J Vector Ecol 38:82–89. doi:10.1111/j.1948-7134.2013.12012.x. 23701611

[18] Bukhari T, Takken W, Koenraadt CJ. 2011. Development of *Metarhizium anisopliae* and *Beauveria bassiana* formulations for control of malaria mosquito larvae. Parasit Vectors 4:23. doi:10.1186/1756-3305-4-23. 21342492 PMC3051916

[19] Valero-Jimenez CA, Debets AJ, van Kan JA, Schoustra SE, Takken W, Zwaan BJ, Koenraadt CJ. 2014. Natural variation in virulence of the entomopathogenic fungus *Beauveria bassiana* against malaria mosquitoes. Malar J 13:479. doi:10.1186/1475-2875-13-479. 25480526 PMC4364330

[20] Howard AF, N’Guessan R, Koenraadt CJ, Asidi A, Farenhorst M, Akogbeto M, Thomas MB, Knols BG, Takken W. 2010. The entomopathogenic fungus *Beauveria bassiana* reduces instantaneous blood feeding in wild multi-insecticide-resistant *Culex quinquefasciatus* mosquitoes in Benin, West Africa. Parasit Vectors 3:87. doi:10.1186/1756-3305-3-87. 20843321 PMC2946288

[21] Lovett B, Bilgo E, Millogo SA, Ouattarra AK, Sare I, Gnambani EJ, Dabire RK, Diabate A, St Leger RJ. 2019. Transgenic *Metarhizium* rapidly kills mosquitoes in a malaria-endemic region of Burkina Faso. Science 364:894–897. doi:10.1126/science.aaw8737. 31147521

[22] Accoti A, Springer Engdahl C, Dimopoulos G. 2021. Discovery of novel entomopathogenic fungi for mosquito-borne disease control. Front Fungal Biol 2:637234. doi:10.3389/ffunb.2021.637234. PMC10512396 37744144

[23] Federici BA. 1995. The future of microbial insecticides as vector control agents. J Am Mosq Control Assoc 11:260–268. 7595459

[24] Coon KL, Brown MR, Strand MR. 2016. Mosquitoes host communities of bacteria that are essential for development but vary greatly between local habitats. Mol Ecol 25:5806–5826. doi:10.1111/mec.13877. 27718295 PMC5118126

[25] Gimonneau G, Tchioffo MT, Abate L, Boissiere A, Awono-Ambene PH, Nsango SE, Christen R, Morlais I. 2014. Composition of *Anopheles coluzzii* and *Anopheles gambiae* microbiota from larval to adult stages. Infect Genet Evol 28:715–724. doi:10.1016/j.meegid.2014.09.029. 25283802

[26] Ramirez JL, Souza-Neto J, Torres Cosme R, Rovira J, Ortiz A, Pascale JM, Dimopoulos G. 2012. Reciprocal tripartite interactions between the *Aedes aegypti* midgut microbiota, innate immune system and dengue virus influences vector competence. PLoS Negl Trop Dis 6:e1561. doi:10.1371/journal.pntd.0001561. 22413032 PMC3295821

[27] Ramirez JL, Short SM, Bahia AC, Saraiva RG, Dong Y, Kang S, Tripathi A, Mlambo G, Dimopoulos G. 2014. *Chromobacterium* Csp_P reduces malaria and dengue infection in vector mosquitoes and has entomopathogenic and in vitro anti-pathogen activities. PLoS Pathog 10:e1004398. doi:10.1371/journal.ppat.1004398. 25340821 PMC4207801

[28] Saraiva RG, Huitt-Roehl CR, Tripathi A, Cheng YQ, Bosch J, Townsend CA, Dimopoulos G. 2018. *Chromobacterium* spp. mediate their anti-*Plasmodium* activity through secretion of the histone deacetylase inhibitor romidepsin. Sci Rep 8:6176. doi:10.1038/s41598-018-24296-0. 29670144 PMC5906607

[29] Saraiva RG, Fang J, Kang S, Anglero-Rodriguez YI, Dong Y, Dimopoulos G. 2018. Aminopeptidase secreted by *Chromobacterium* sp. Panama inhibits dengue virus infection by degrading the E protein. PLoS Negl Trop Dis 12:e0006443. doi:10.1371/journal.pntd.0006443. 29694346 PMC5937796

[30] Short SM, van Tol S, MacLeod HJ, Dimopoulos G. 2018. Hydrogen cyanide produced by the soil bacterium *Chromobacterium* sp. Panama contributes to mortality in *Anopheles gambiae* mosquito larvae. Sci Rep 8:8358. doi:10.1038/s41598-018-26680-2. 29844510 PMC5974309

[31] Short SM, van Tol S, Smith B, Dong Y, Dimopoulos G. 2018. The mosquito adulticidal *Chromobacterium* sp. Panama causes transgenerational impacts on fitness parameters and elicits xenobiotic gene responses. Parasit Vectors 11:229. doi:10.1186/s13071-018-2822-8. 29622036 PMC5887189

[32] Caragata EP, Otero LM, Carlson JS, Borhani Dizaji N, Dimopoulos G. 2020. A nonlive preparation of *Chromobacterium* sp. Panama (Csp_P) is a highly effective larval mosquito biopesticide. Appl Environ Microbiol 86:e00240-20. doi:10.1128/AEM.00240-20. 32220845 PMC7237781

[33] Mouhamadou CS, de Souza SS, Fodjo BK, Zoh MG, Bli NK, Koudou BG. 2019. Evidence of insecticide resistance selection in wild *Anopheles coluzzii* mosquitoes due to agricultural pesticide use. Infect Dis Poverty 8:64. doi:10.1186/s40249-019-0572-2. 31307509 PMC6631620

[34] Nkya TE, Poupardin R, Laporte F, Akhouayri I, Mosha F, Magesa S, Kisinza W, David JP. 2014. Impact of agriculture on the selection of insecticide resistance in the malaria vector *Anopheles gambiae*: a multigenerational study in controlled conditions. Parasit Vectors 7:480. doi:10.1186/PREACCEPT-1961934091135119. 25318645 PMC4201709

[35] Riaz MA, Chandor-Proust A, Dauphin-Villemant C, Poupardin R, Jones CM, Strode C, Regent-Kloeckner M, David JP, Reynaud S. 2013. Molecular mechanisms associated with increased tolerance to the neonicotinoid insecticide imidacloprid in the dengue vector *Aedes aegypti*. Aquat Toxicol 126:326–337. doi:10.1016/j.aquatox.2012.09.010. 23058251

[36] David MR, Garcia GA, Valle D, Maciel-de-Freitas R. 2018. Insecticide resistance and fitness: the case of four *Aedes aegypti* populations from different Brazilian regions. Biomed Res Int 2018:6257860. doi:10.1155/2018/6257860. 30402487 PMC6198578

[37] Freeman JC, Smith LB, Silva JJ, Fan Y, Sun H, Scott JG. 2021. Fitness studies of insecticide resistant strains: lessons learned and future directions. Pest Manag Sci 77:3847–3856. doi:10.1002/ps.6306. 33506993

[38] Carvalho KDS, Guedes DRD, Crespo MM, de Melo-Santos MAV, Silva-Filha M. 2021. *Aedes aegypti* continuously exposed to *Bacillus thuringiensis* svar. *israelensis* does not exhibit changes in life traits but displays increased susceptibility for Zika virus. Parasit Vectors 14:379. doi:10.1186/s13071-021-04880-6. 34321098 PMC8317411

[39] David JP, Faucon F, Chandor-Proust A, Poupardin R, Riaz MA, Bonin A, Navratil V, Reynaud S. 2014. Comparative analysis of response to selection with three insecticides in the dengue mosquito *Aedes aegypti* using mRNA sequencing. BMC Genomics 15:174. doi:10.1186/1471-2164-15-174. 24593293 PMC4029067

[40] Despres L, Stalinski R, Tetreau G, Paris M, Bonin A, Navratil V, Reynaud S, David JP. 2014. Gene expression patterns and sequence polymorphisms associated with mosquito resistance to *Bacillus thuringiensis israelensis* toxins. BMC Genomics 15:926. doi:10.1186/1471-2164-15-926. 25341495 PMC4223840

[41] Carvalho KDS, Crespo MM, Araujo AP, da Silva RS, de Melo-Santos MAV, de Oliveira CMF, Silva-Filha M. 2018. Long-term exposure of *Aedes aegypti* to *Bacillus thuringiensis* svar. *israelensis* did not involve altered susceptibility to this microbial larvicide or to other control agents. Parasit Vectors 11:673. doi:10.1186/s13071-018-3246-1. 30594214 PMC6311009

[42] Ye YH, Chenoweth SF, McGraw EA. 2009. Effective but costly, evolved mechanisms of defense against a virulent opportunistic pathogen in *Drosophila melanogaster*. PLoS Pathog 5:e1000385. doi:10.1371/journal.ppat.1000385. 19381251 PMC2663048

[43] Atyame CM, Alout H, Mousson L, Vazeille M, Diallo M, Weill M, Failloux AB. 2019. Insecticide resistance genes affect *Culex quinquefasciatus* vector competence for West Nile virus. Proc R Soc B 286:20182273. doi:10.1098/rspb.2018.2273. PMC6367175 30963855

[44] Ndo C, Kopya E, Irving H, Wondji C. 2019. Exploring the impact of glutathione *S*-transferase (GST)-based metabolic resistance to insecticide on vector competence of *Anopheles funestus* for *Plasmodium falciparum*. Wellcome Open Res 4:52. doi:10.12688/wellcomeopenres.15061.1. 31976375 PMC6957023

[45] Alout H, Ndam NT, Sandeu MM, Djegbe I, Chandre F, Dabire RK, Djogbenou LS, Corbel V, Cohuet A. 2013. Insecticide resistance alleles affect vector competence of *Anopheles gambiae* s.s. for *Plasmodium falciparum* field isolates. PLoS One 8:e63849. doi:10.1371/journal.pone.0063849. 23704944 PMC3660590

[46] Despres L, Stalinski R, Faucon F, Navratil V, Viari A, Paris M, Tetreau G, Poupardin R, Riaz MA, Bonin A, Reynaud S, David JP. 2014. Chemical and biological insecticides select distinct gene expression patterns in *Aedes aegypti* mosquito. Biol Lett 10:20140716. doi:10.1098/rsbl.2014.0716. 25540155 PMC4298186

[47] Sim S, Jupatanakul N, Ramirez JL, Kang S, Romero-Vivas CM, Mohammed H, Dimopoulos G. 2013. Transcriptomic profiling of diverse *Aedes aegypti* strains reveals increased basal-level immune activation in dengue virus-refractory populations and identifies novel virus-vector molecular interactions. PLoS Negl Trop Dis 7:e2295. doi:10.1371/journal.pntd.0002295. 23861987 PMC3701703

[48] Poole-Smith BK, Hemme RR, Delorey M, Felix G, Gonzalez AL, Amador M, Hunsperger EA, Barrera R. 2015. Comparison of vector competence of *Aedes mediovittatus* and *Aedes aegypti* for dengue virus: implications for dengue control in the Caribbean. PLoS Negl Trop Dis 9:e0003462. doi:10.1371/journal.pntd.0003462. 25658951 PMC4319915

[49] Love MI, Huber W, Anders S. 2014. Moderated estimation of fold change and dispersion for RNA-seq data with DESeq2. Genome Biol 15:550. doi:10.1186/s13059-014-0550-8. 25516281 PMC4302049

[50] Global Malaria Program. 2018. Test procedures for insecticide resistance monitoring in malaria vector mosquitoes. World Health Organization, Geneva, Switzerland. https://apps.who.int/iris/bitstream/handle/10665/250677/9789241511575-eng.pdf.

[51] Abbott WS. 1987. A method of computing the effectiveness of an insecticide. 1925. J Am Mosq Control Assoc 3:302–303. 3333059

